# Do fluoroquinolones increase aortic aneurysm or dissection incidence and mortality? A systematic review and meta-analysis

**DOI:** 10.3389/fcvm.2022.949538

**Published:** 2022-08-09

**Authors:** Can Chen, Benjamin Patterson, Ruan Simpson, Yanli Li, Zhangzhang Chen, Qianzhou Lv, Daqiao Guo, Xiaoyu Li, Weiguo Fu, Baolei Guo

**Affiliations:** ^1^Department of Pharmacy, Zhongshan Hospital, Fudan University, Shanghai, China; ^2^Department of Vascular Surgery, University Hospital Southampton, Southampton, United Kingdom; ^3^Department of Pathology, Portsmouth Hospitals NHS Trust, United Kingdom; ^4^Department of Vascular Surgery, Zhongshan Hospital, Institute of Vascular Surgery, Fudan University, Shanghai, China; ^5^National Clinical Research Center for Interventional Medicine, Shanghai, China; ^6^Qingpu Branch of Zhongshan Hospital, Fudan University, Shanghai, China

**Keywords:** fluoroquinolones, aortic aneurysm, aortic dissection, systematic review, meta-analysis

## Abstract

**Objective:**

The aim of this study was to determine the association between fluoroquinolones (FQs) use, the risk of *de novo* aortic aneurysm or dissection (AAD), and the prognosis of patients with pre-existing AAD.

**Materials and methods:**

We searched PubMed, EMBASE, CENTRAL, Scopus, and Web of Science on 31 March 2022. Observational studies that evaluated the association of FQs with AAD risk in the general population or FQs with the prognosis of patients with preexisting AAD and presented adjusted effect estimates were included. Two reviewers assessed study eligibility, extracted data, and assessed the risk of bias and certainty of evidence using GRADE.

**Results:**

Of the 13 included studies, 11 focused on the association of FQs with *de novo* AAD incidence, and only one study investigated the association of FQs with the patient with AAD prognosis. FQ use was associated with an increased risk of *de novo* AAD within 30 days (RR: 1.42; 95% CI: 1.11–1.81; very low certainty) and 60 days (RR: 1.44; 95% CI: 1.26–1.64; low certainty). Specifically, the association was significant when compared with amoxicillin, azithromycin, doxycycline, or no antibiotic use. Furthermore, patients with preexisting AAD exposure to FQ had an increased risk of all-cause mortality (RR: 1.61; 95% CI: 1.50–1.73; moderate certainty) and aortic-specific mortality (RR: 1.80; 95% CI: 1.50–2.15; moderate certainty), compared to the non-exposed FQ group within a 60-day risk period.

**Conclusion:**

FQs were associated with an increased incidence of AAD in the general population and a higher risk of adverse outcomes in patients with preexisting AAD. Nevertheless, the results may be affected by unmeasured confounding factors. This should be considered by physicians contemplating using FQs in patients with aortic dilation and those at high risk of AAD.

**Systematic Review Registration:**

[https://www.crd.york.ac.uk/prospero/], identifier [CRD42021230171].

## Introduction

Fluoroquinolones (FQs) are one of the most commonly used classes of antibiotics, partly due to their wide activity spectrum, excellent bioavailability, extensive penetration of tissue, and successful microbiological outcomes (1, 2). Although FQs are well tolerated (3, 4), it has been suggested that they might exacerbate collagen-associated diseases owing to collagen loss and tissue degeneration (5–8).

Type I and type III collagen comprise the majority (80–90%) of collagen in the aorta (9), and FQs may contribute to the development of aortic disease (10, 11). Despite this plausible link, studies to characterize this relationship have yielded conflicting results. Several studies (12–17) using large administrative datasets found that recent FQs exposure was strongly associated with an increased risk of AAD, but after adjusting for the comparator antibiotics, two recent studies (18, 19) did not support this finding. Although a consensus on whether FQ causes *de novo* aortic disease has not yet been reached (20, 21), this potential association has raised several other important clinical questions, particularly regarding whether FQs can precipitate aortic complications in patients with existing aortic disease. It has been shown that FQ exposure could increase the risk of acute aortic dissection or rupture for patients with underlying aortopathy (10, 11, 22). Subsequently, Chen et al. (23) found that FQ exposure in patients with preexisting AAD was associated with a higher risk of adverse outcomes relative to non-exposure.

Although aortic events associated with FQs are thought to be rare (15), the United States alone has approximately 14 million annual FQ prescriptions (24, 25), and 20% of patients with AAD receive FQs during their hospitalization (26). Considering the serious adverse outcomes associated with aortopathy, including death, FQ-associated aortopathy constitutes a major health problem worldwide. We analyzed the association between FQs and the incidence and prognosis of AAD by systematic review and meta-analysis.

## Materials and methods

This systematic review and meta-analysis followed the recommendations (27) and was reported in accordance with the Preferred Reporting Items for Systematic Reviews and Meta-Analyses (PRISMA) statement (28). The review was registered through PROSPERO with the registration number CRD42021230171.

## Data sources and searches

We searched PubMed, EMBASE, the Cochrane Central Register of Controlled Trials, Scopus, and Web of Science on 31 March 2022, for studies on the association between FQs and the risk of AAD incidence, all-cause mortality, and aortic-specific mortality in patients with AAD. In addition, the reference lists of relevant publications were reviewed to identify any additional studies that met the eligibility criteria. The electronic search strategy is presented in the ESM.

## Study selection and eligibility criteria

Studies were included if the following criteria were met: (1) population: participants who were initially free of AAD and did not have an AAD history when they entered a cohort that was subsequently followed to determine the risk of AAD incidence, or patients with AAD who were enrolled in a cohort that was followed to determine the risk of aortic-specific mortality or all-cause mortality; (2) definition of FQ exposure: participant received at least one prescription or a reimbursement for FQs; (3) studies reported the risk estimates of AAD incidence or mortality exposed to FQs vs. no FQs. Studies that did not report multivariable-adjusted estimates for at least one of the outcomes of interest, reported as abstracts only, presented without any data on relevant outcomes, or included data from Vigibase or the US Food and Drug Administration Adverse Event Reporting System (FAERS) database were excluded. Two reviewers (C.C. and B.G.) independently and in duplicate screened titles and abstracts, followed by full-text screening of potentially eligible studies. Discrepancies were solved through consensus or by the involvement of a third reviewer (W.F.).

The outcomes were the risk of AAD incidence in general populations and the risk of aortic-specific mortality, or all-cause mortality, in patients with AAD, within 30-, 60-, and 90-day risk periods following FQ exposure.

## Data extraction, risk of bias assessments, and certainty of evidence

We extracted the following data from eligible studies: (1) study characteristics (first author, publication year, country, data source, definition of FQs exposure, and comparators); (2) population characteristics (age, gender, and number of participants); and (3) outcomes (number of events, exposure to FQs risk period, and the variables used for adjustment).

We assessed the risk of bias in the studies using the Quality in Prognosis Studies (QUIPS) tool (29). We categorized studies as having an overall low risk of bias if they had 5 or 6 low risk of bias domains, an overall high risk of bias if they had 2 or more high risk of bias domains, and an overall moderate risk of bias if they had all other studies (30).

The certainty of the evidence was assessed as high, moderate, low, or very low using the Grading of Recommendations Assessment, Development, and Evaluation (GRADE) approach adapted to prognosis studies (31). This assessment was based on risk of bias, consistency, precision, directness, and other concerns including publication bias (32), where two reviewers (C.C. and B.G.) independently, and in duplicate, extracted data, assessed risk of bias, and assessed certainty of evidence using GRADE. Any discrepancies were resolved by consensus or through the involvement of a third reviewer (W.F.).

## Data synthesis and statistical analyzes

We performed a meta-analysis by extracting and pooling adjusted relative effect estimates (27, 33). When merging data from studies that reported only an odds ratio (OR), we treated the OR as an RR (34) because the AA annual incidence is low (0.4–0.67%) (35). Studies comparing FQs to various controls, including other, or no antibiotics were included in the meta-analysis by making multiple pair-wise comparisons between all possible intervention group pairs (33). Statistical heterogeneity was addressed through the consistency of point estimates and the extent of CIs overlapped (36). We conducted subgroup analyzes according to age, sex, study type, comparators, ruptured or unruptured AA/AD, type of aortic disease, or anatomical site. In the sensitivity analyzes, we restricted analysis to AAD patients with baseline imaging to minimize potential surveillance bias and to individuals with infections to reduce selection bias. For outcomes reported by two or more studies, sensitivity analyzes were performed using a fixed effects model and individually excluded studies to explore the impact of each study on the overall results. For studies that gathered data from the same database and reported similar outcomes, recent publications were selected for primary analysis, and early publications were selected for sensitivity analysis. All meta-analyzes were conducted using Review Manager 5.4. The probability of publication bias was tested by means of Egger’s test using Stata 14.0 software. Two-sided *P*-values < 0.05 and 95% CIs, not including 1.00, were considered as statistically significant.

## Results

### Study selection and characteristics

**FIGURE 1:**
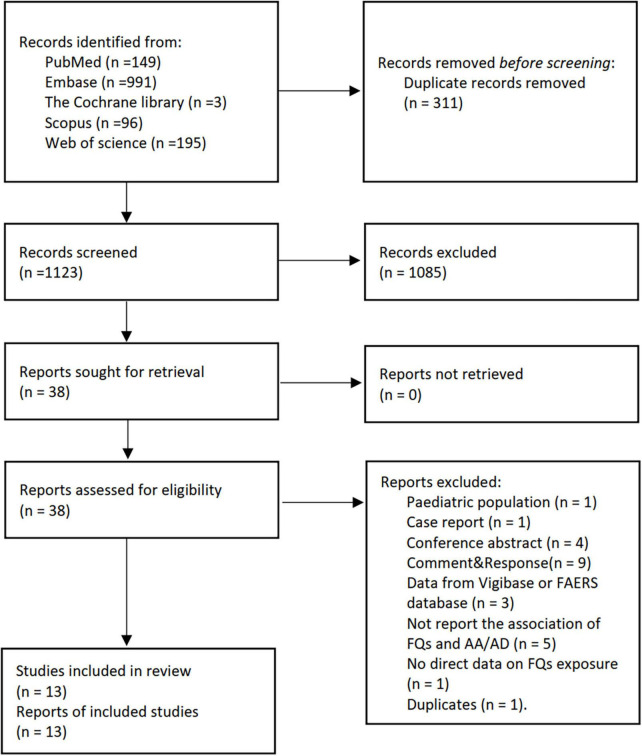
The PRISMA flow diagram for literature screening. FQs, fluoroquinolones; AA, aortic aneurysm; AD, aortic dissection.

We screened 1,434 abstracts and 38 full-text papers and included 13 studies (12–19, 23, 37–40) (Figure 1 and Supplementary Table 1). A total of 11 studies (12–19, 37–40) focused on the association of FQs with *de novo* AAD incidence, and only one study (23) investigated the association of FQs with all-cause mortality and aortic-specific mortality of patients with existing AAD. Six studies (12, 15, 17, 19, 23, 40) were cohort studies, three (13, 18, 39) were nest case-control studies, and four (14, 16, 37, 38) were self-controlled studies, including two case-time-control studies (14, 16), one case-crossover studies (38), and one self-controlled case series studies (37). Five studies (13, 14, 18, 23, 40) were from Taiwan, three (17, 19, 37) from the United States, and the others were from Canada (12), Sweden (15), France (16), Denmark (38), and Korea (39). Data used in all studies were obtained from various administrative healthcare databases across countries. Seven studies (13, 14, 17, 18, 23, 37, 40) limited inclusion to patients aged 18 years or older of which two (18, 23) applied an age limit of 20 years, four (15, 19, 38, 39) included 40 years or older, and one (12) included 65 years or older. The definition of FQ exposure in all studies relied on the occurrence of a FQ prescription or reimbursement. The identification of AAD in all studies was based on the ICD-9 or ICD-10 codes with, or without, advanced imaging. There was substantial variation in the analytic strategies used, including the adjusted variables.

The risk of bias was low in 4 studies and moderate in 9 studies. The bias mainly came from the outcome measurement and study of confounding domains (Supplementary Tables 1–13 in the ESM). Additional study characteristics are shown in Table 1 and Supplementary Tables 1–13 in the ESM.

**TABLE 1:** Characteristics of included studies.

Authors	Year	Region	Study type	Population	Intervention and control	Age (years)*	Sex, male (%)	Risk period
**FQs and the risk of AAD incidence**								
Newtonet al. (17)	2021	United States	Cohort study	Adults aged 18 to 64 years	I: Oral FQ C: amox-clav, azithromycin, cephalexin, clindamycin, and SMX-TMP	I: 47 (36–57); C: 43 (31–54)	I: 38.7%; C: 40.5%	90 days
Gopalakrishnan et al. (19)	2020	United States	Cohort study	Patients ≥ 50 years diagnosed with pneumonia or UTI	Pneumonia cohort: I: FQ; C: azithromycin; UTI cohort: I: FQ; C: SMX-TMP; Amoxicillin cohort: I: FQ; C:Amoxicillin;	Pneumonia cohort: I: 63.68 ± 10.93; C: 63.63 ± 10.92; UTI cohort: I: 62.07 ± 10.36; C: 62.04 ± 10.30; Amoxicillin cohort: I: 60.55 ± 9.34; C: 60.68 ± 9.28;	Pneumonia cohort: I: 46.4%; C: 46.3% UTI cohort: I: 13.3%; C:13.0% Amoxicillin cohort: I: 44.0%; C: 44.0%	60 days
Pasternak et al. (15)	2018	Sweden	Cohort study	Patients ≥ 50 years	I: FQ; C:Amoxicillin;	I: 47 (36-57); C: 43 (31–54)	I: 45.0%; C: 45.0%	60 days
Daneman et al. (12)	2015	Canada	Cohort study	Adults aged 65	I: FQ; C: No FQ**;	I: 65 C: 65	I: 51.4%; C: 51.1%	30 days
Dong et al. (18)	2020	Taiwan	Nested case-control study	Patients ≥ 20 years	I: FQ; C1: Amox-clav or amp-sulb C2: Extended-spectrum ceph	AAD patients: 67.41 ± 15.03; Matched controls patients: 67.41 ± 15.03	AA/AD patients: 71.4%; Matched controls: 71.4%	30, 60 days
Lee et al. (13)	2015	Taiwan	Nested case-control	Adults’ patients diagnosed with AAD.	I: FQ; C: No FQ**;	AA/AD patients: 74.17 ± 11.7(AA)/ 66.2 ± 14.5(AD); Matched controls patients: 71.0 ± 13.7	AA/AD patients: 74.1%(AA)/71.5%(AD); Matched controls patients: 72.9%	60, 61–365 days
Lawaetz Kristensen et al. (38)	2021	Denmark	Case-crossover study	Patients ≥ 50 years diagnosed with ruptured AA	I: FQ; C: No FQ**;	FQ group: 77 (70–81); Control group: 77 (71–82)	FQ group, 69.0%; Control group, 66.5%	28, 60, 90 days
Aspinall et al. (37)	2020	United States	Self-controlled case series analysis study	Veterans ≥ 18 years who had the outcomes of AAD	I: FQ; C1: Amoxicillin; C2: Azithromycin; C3: Cefuroxime/cephalexin; C5: Doxycycline; C6: SMX-TMP; C7: No antibiotics	FQ group, 68.6 ± 8.8; Control group, NR.	FQ group, 98.3%; Control group, NR.	30, 60 days
Maumus-Robert et al. (16)	2019	France	Case-time-control study	Patients ≥ 18 years with incident aortoiliac aneurysm or dissection	I: FQ; C1: Amoxicillin	FQ group, 70 (62–80); Amoxicillin group, 67 (57–79)	FQ group, 64.0%; Amoxicillin group, 74.7%	30, 60, 90 days
Lee et al. (14)	2018	Taiwan	Case-crossover analysis/control-crossover (case-time-control analysis)	Inpatients diagnosed with AAD	I: FQ; C: No FQ**	AAD patients, 70.58 ± 13.77; Matched controls patients without AAD, 70.48 ± 13.69.	AA/AD patients, 72.46%; Matched controls patients without AA/AD, 72.46%	60, 120, 180 days
Son et al. (39)	2022	Korea	Nested case–control study	Patients ≥ 40 years diagnosed with AAD	I: FQ; C: No FQ**	NR	AAD 62.5% Matched controls 62.5%	60 days
Chen et al. (40)	2022	Taiwan	Cohort study	Patients diagnosed with urinary tract infections.	I: FQ; C: Cephalosporins	NR	I: 28.2%; C: 28.1%	90 days
**FQs and mortality of patients with AAD**								
Chen et al. (23)	2021	Taiwan	Cohort study	Patients who were admitted for AAD	I: FQ; C1: No FQ**; C2: Amoxicillin C2: No amoxicillin**	FQ group, 73.1 (63.4–79.9); control group (No FQ): 68.5 (55.7–77.3); Amoxicillin group, 67.2 (55.2–76.0); control group (No Amoxicillin): 68.7 (55.9–77.4).	FQ group, 69.7%; control group (No FQ):72.0%; Amoxicillin group, 74.5%; control group (No Amoxicillin): 71.9%.	60 days

*Present as mean ± standard deviation or median (interquartile range). **No antibiotics or other antibiotics. FQ, fluoroquinolone; AAD, aortic aneurysm or dissection; AA, aortic aneurysm; AD, aortic dissection; I, intervention; C, control; UTI, urinary tract infection.

### Synthesis of results

#### Fluoroquinolones (FQs) and the risk of aortic aneurysm or dissection (AAD) incidence

**FIGURE 2:**
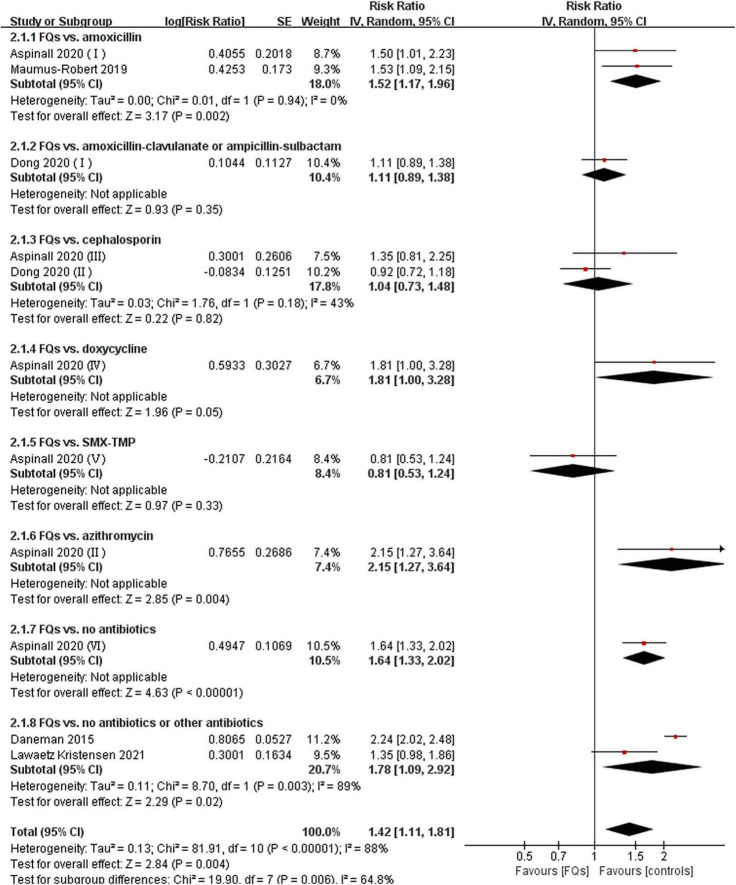
Forest plot of the risk of aortic aneurysm or dissection (AAD) in the comparison of fluoroquinolones (FQs) vs. controls within a 30-day risk period. FQs, fluoroquinolones; SMX-TMP, combined trimethoprim and sulfamethoxazole; IV, inverse variance; CI, confidence interval.

**FIGURE 3:**
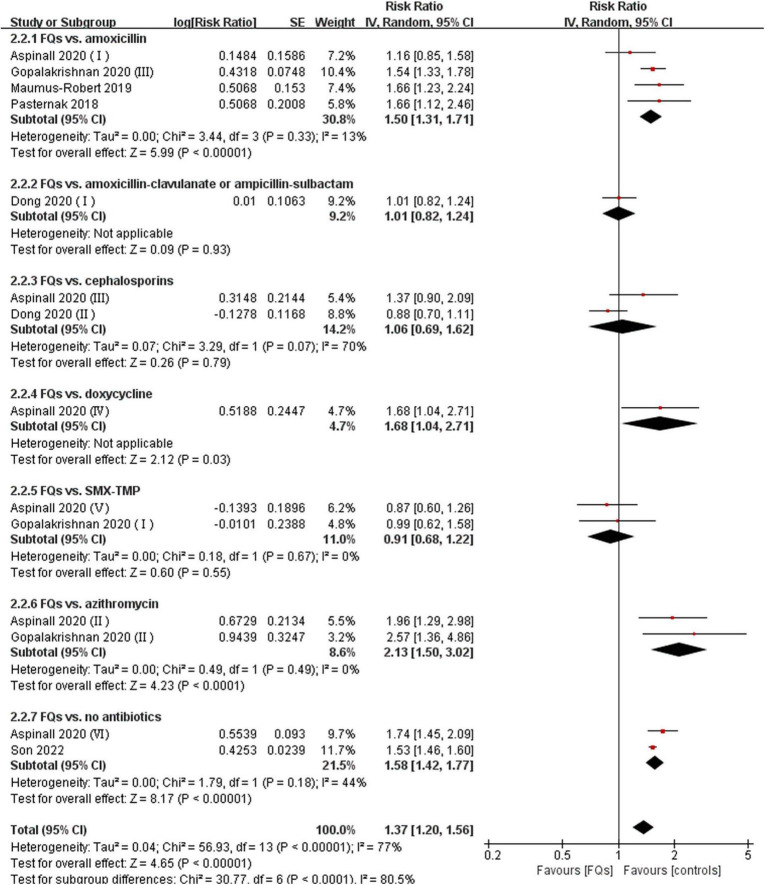
Forest plot of the risk of aortic aneurysm or dissection (AAD) in the comparison of fluoroquinolones (FQs) vs. controls within a 60-day risk period. FQs, fluoroquinolones; SMX-TMP, combined trimethoprim and sulfamethoxazole; IV, inverse variance; CI, confidence interval.

**FIGURE 4:**
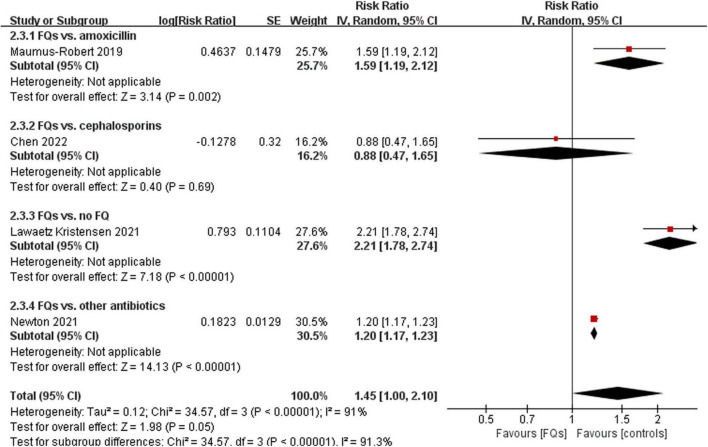
Forest plot of the risk of aortic aneurysm or dissection (AAD) in the comparison of fluoroquinolones (FQs) vs. controls within a 90-day risk period. FQs, fluoroquinolones; IV, inverse variance; CI, confidence interval.

There were four (12, 18, 37, 38), eight (13, 15, 16, 18, 19, 37–39), and four (16, 37, 38, 40) studies that linked the association of FQ use with the incidence of *de novo* AAD within 30-, 60-, and 90-day risk windows, respectively. The meta-analysis indicated that FQs were associated with an increased *de novo* AAD risk at 30-day (RR: 1.42; 95% CI: 1.11–1.81; very low certainty) and 60-day (RR: 1.44; 95% CI: 1.26–1.64; low certainty) risk window. AAD incidence showed a trend to increase after exposure to FQs within 90 days, but the difference did not reach statistical significance (RR: 1.45; 95% CI: 1.00–2.10; low certainty). When stratified by comparators, the association of FQs with AAD risk was significantly different across various comparators at 30-day (*P* = 0.006), 60-day (*P* < 0.001), and 90-day (*P* < 0.001) risk periods. Within any risk period, FQs were only associated with higher AAD risk when compared with amoxicillin, azithromycin, or no antibiotics and had a higher AAD rate compared with doxycycline in a 60-day risk period. The forest plots of the meta-analyzes are displayed in Figures 2–4, with different comparator subgroups also shown separately. The summary estimates of the FQ association with AAD risk are presented in Table 15.

When analyzing the AA and AD patients separately, the effect of FQs on AD and AA risks was not significantly different (Supplementary Figures 1A,B). When stratified by study type, the association of FQs with the incidence of AAD varied significantly across various study designs. Specifically, within a 30-day risk period (self-control studies: RR: 1.46; 95% CI: 1.23–1.73; cohort studies: RR: 2.24; 95% CI: 2.02–2.48; nest case-control studies: RR: 1.02; 95% CI: 0.85–1.22; *P* < 0.001); within a 90-day risk period (self-control studies: RR: 1.90; 95% CI: 1.38–2.62; cohort studies: RR: 1.20; 95% CI: 1.17–1.23; *P* = 0.005). However, the association was not significantly different across various study designs within a 60-day risk period (self-control studies: RR: 1.64; 95% CI: 1.30–2.06; cohort studies: RR: 1.56; 95% CI: 0.96–2.56; nest case-control studies: RR: 1.34; 95% CI: 0.95–1.89; *P* = 0.64). Minimal differences were observed when stratified by age, sex, incidence of ruptured or unruptured AA or AD, or anatomical site (Supplementary Tables 16–18).

In the sensitivity analysis, we explored the comparison of FQ and controls with analysis restricted to patients with AAD with baseline imaging and found that the association was attenuated (RR: 1.05; 95% CI: 0.94–1.18). Results were similar when the analysis was restricted to individuals with infection (Supplementary Table 19). The sensitivity analysis performed *via* a fixed effects model did not change appreciably when compared to the primary analyzes. Furthermore, we performed sensitivity analyzes by excluding each study individually from the primary analyzes, and the outcomes remained unchanged. The two studies (13, 14) reported using the same database and had the same outcomes within 60 days, and hence the sensitivity analysis results were similar to those of the primary analysis.

#### Fluoroquinolones (FQs) and mortality of aortic aneurysm or dissection (AAD) patients

**FIGURE 5:**
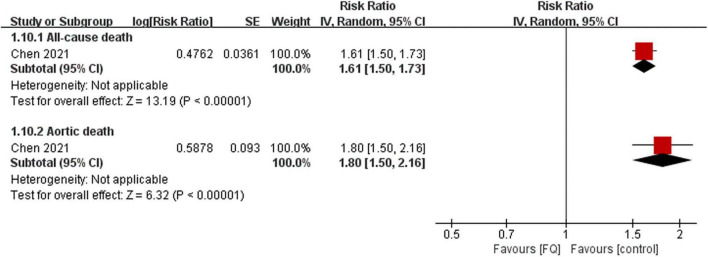
Forest plot of mortality risk of aortic aneurysm or dissection (AAD) patients in the comparison of fluoroquinolones (FQs) vs. control within a 60-day risk period. FQs, fluoroquinolones; IV, inverse variance; CI, confidence interval.

Only one study (23) investigated whether the use of FQs increases the 60-day risk of mortality in the AAD population. The findings suggest that exposure to FQs is associated with a higher risk of all-cause mortality (RR: 1.61; 95% CI: 1.50–1.73; moderate certainty) and aortic-specific mortality (RR: 1.80; 95% CI: 1.50–2.15; moderate certainty) compared with non-FQs (Figure 5, Supplementary Table 20). However, patients with AAD exposed to amoxicillin were not significantly associated with any risk outcomes compared with non-amoxicillin antibiotics (Supplementary Table 21). By comparing the risk of outcomes between the FQ and amoxicillin exposure periods for the same patient, the results demonstrated that the mortality risks were significantly higher during the FQ exposure period than during the amoxicillin exposure period (Supplementary Table 22). The summary estimates for the association of FQs and the AAD patient prognosis are presented in Supplementary Table 23.

When analyzing the AA and AD patients separately, the effect of FQs did not differ significantly between the AD and AA groups (Supplementary Table 24).

#### Publication bias

When analyzing the association of FQ use with the *de novo* AAD incidence within 30-day (*P* = 0.37), 60-day (*P* = 0.68), and 90-day (*P* = 0.51) risk windows, the Egger’s test indicated that no evidence of publication bias was found.

## Discussion

Early studies (12–15) suggesting a possible association between FQs use and the risk of AAD, combined with reasonable evidence of a mechanistic link with other collagen-related diseases disorders (7, 8), led to the US Food and Drug Administration (FDA) (41) issuing a warning against FQ use in patients who were at risk of aortic disease. This study adds to the evidence that FQ use is associated with an increased risk of *de novo* AAD within a 90-day risk window. Patients with preexisting AAD and exposure to FQ had an increased risk of all-cause mortality and aortic-specific mortality relative to controls within a 60-day risk period, adding further weight to the FDA warning, which was based on inferences from studies designed for the general population rather than specific high-risk groups.

Although the use of FQs appears to lead to a higher risk of AAD incidence than no use of FQs, we found that the associations between FQs and AAD risk may change with various comparator antibiotics. Specifically, the associations were only significant when compared with antibiotics with a broad antimicrobial profile. Patients who are prescribed a broad-spectrum antibiotic potentially have more severe infections than those prescribed narrow-spectrum antibiotics or no antibiotics. Since severe infection may also independently damage the aortic endothelium, the infection may itself increase the risk of AAD (18). Another reason is that patients with more severe infections or indications for FQs therapy are more likely to receive an imaging examination, leading to a higher ascertainment rate (42, 43). Consequently, if the analysis was restricted to patients with prior imaging, then the association was attenuated, probably due to decreased ascertainment of “new” disease. These biases may confound the apparent association between FQs and AAD incidence. Additionally, Newton et al. (17) considered that this inconsistency may be due to the different analytical approaches employed in the studies, such as the inclusion of different populations, different definitions of FQ exposure, different sample sizes, and different ways to adjust confounding factors.

While there is limited evidence for a mechanistic link between antimicrobial use and aortic disease in humans, LeMaire et al. (22) found that wild-type mice given a high-fat diet, angiotensin II infusion, and exposure to ciprofloxacin experienced more severe aortic wall degeneration and a higher incidence of aortic aneurysm, dissection, and rupture, compared with test control mice. Another study (11) revealed that ciprofloxacin-treated mice suffered accelerated aortic enlargement and an increased incidence of aortic dissection and rupture, compared with vehicle-treated mice. This suggests that ciprofloxacin should be avoided in patients with Marfan syndrome. Campana et al. (11, 44) reported a patient who presented fever of unknown origin, elevated systemic inflammation markers, and radiological evidence of aortitis and pneumonia, who was subsequently treated with levofloxacin. Aortic rupture then occurred after 5 days after levofloxacin therapy was commenced. It was postulated that FQs could trigger the activation of ECM remodeling and an increase in MMP activity as a potential mechanism for this effect. Guzzardi et al. (44, 45) published a report suggesting that patients with alpha-1 antitrypsin (A1AT) deficiency and longstanding FQ use (26 months) may have a higher risk of AAD, although patients with A1AT deficiency are not typically considered at risk of aortopathy despite maladaptive connective tissue changes.

The suppression of extracellular matrix (ECM) biosynthesis and stability and the induction of ECM degradation may be key mechanisms underlying the effects of FQ on aortic destruction, dissection, and rupture (22). Consistent with that reported in the cornea (46, 47), tendon cells and tissues (48), and fibroblasts (49), ciprofloxacin exposure significantly increased MMP-2 and MMP-9 expression in the aortic wall, facilitating increased ECM destruction (11, 22). Guzzardi et al. (10) showed that FQ exposure induced a proteolytic MMP-TIMP imbalance driven by decreased TIMP expression, while simultaneously attenuating collagen expression in human aortic myofibroblast mediated ECM dysregulation. Furthermore, ciprofloxacin decreases the expression of Lysyl oxidase (LOX) (22), which is critical in elastic fiber assembly and stabilization and plays an important role in maintaining the integrity of aortic wall. In addition, the activation of stimulator of interferon genes (STING) (50–52), a pro-inflammatory cytosolic DNA sensor that plays a critical role in aortic degeneration, dissection, and rupture (53), is involved in the ciprofloxacin-induced suppression of LOX expression and the induction of MMP expression. Ciprofloxacin may also cause aortic destruction by increasing the number of TUNEL-positive cells in the aortic wall and inducing cell death in cultured aortic SMCs. This finding, corroborated by other studies, shows that ciprofloxacin induces cytotoxicity and death in various types of cells such as tenocytes (50, 51), lens epithelial cells (54), chondrocytes (55), and osteoblasts (56).

There are several potentially important implications of this study. FQs are commonly used in the empirical treatment of infected AAs to cover *Salmonella* species and sensitive *Staphylococcus*, which are highly prevalent bacterial species in this setting (57, 58). Alternate antibiotics should be sought as this could potentially increase the risk of aortic rupture in this already high-risk group. Furthermore, a retrospective cohort study (26) revealed that a large number of AAD patients received FQs during hospital admissions in general. Stronger evidence of the association of FQs with aortic-related adverse events may help inform enhanced antimicrobial stewardship around their use, especially given that more than 20% of patients with FQs may not have an appropriate indication for their use (17). Finally, for high-risk patients who have no choice but to use FQs, appropriate surveillance will be needed to minimize the hazard of aortic adverse events.

Despite these associations, additional research is needed to prove a causal link between FQs and aortic events. An *in vitro* study (10) showed that higher doses for longer periods may increase the susceptibility to FQ-associated acute aortic events. Clinical studies investing in the dose–response relationship between FQ use and the risk of aortic-related adverse events may be warranted. Furthermore, since FQ exposure might have different effects on cells in patients with variable comorbidities, clinical phenotypes, and connective tissue defects (10), clinical studies are needed to clarify differences between these subpopulations. This review only included one study (23) on the association of FQs and the mortality of AAD patients, and from this, it was difficult to determine whether FQ exposure conferred poor prognosis to AAD patients compared with other broad-spectrum antibiotics. Third-generation FQs do not appear to be associated with an increased risk of Achilles tendon rupture compared with first- and second-generation FQs and non-FQs (59), but the potential effect on AAD needs further study.

This systematic review has several strengths. First, the datasets used in this analysis may have contained overlapping patients, and thus complete independence between the databases cannot be ascertained. By reporting the association between FQ use and aortic events grouped by different risk periods (30, 60, or 90 days), this study effectively avoided data merging from studies using the same database. Second, compared with previously published meta-analyses (60–63), this review added to the current literature in studying the relationship between FQs and AAD mortality, and conducted more detailed subgroup analyzes to explore differences in the effects of FQ across different subgroups, and indeed found that differences in the selection of comparators had a substantial effect on the results.

Limitations include that the AAD diagnosis was according to ICD-9/10 codes with or without baseline advanced imaging examination, which is less accurate than clinical adjudication. This implies that some undiagnosed aneurysms were not identified, and aneurysm size or indications for surgical intervention were unavailable. Furthermore, since abdominal imaging was not routinely performed, it is possible that aneurysms classified as incidental may have been preexisting, or FQs simply aggravated a preexisting condition rather than initiated a *de novo* aneurysm. Second, as with all administrative databases, there is no available information on adherence to prescriptions. Therefore, the possibility of exposure misclassification cannot be excluded. Some included studies suggest that such exposure misclassification was random and did not bias the results (14, 17). Third, systematic reviews and meta-analyzes of observational studies are extremely sensitive to biases resulting from confounding factors. Although adjustment for numerous covariates was conducted, unknown confounders may have caused residual confounding. For instance, several risk factors associated with aneurysm development, such as smoking, were not captured (or reliably captured) in the claims data. Zhang et al. (64) quantified the potential influence of unmeasured confounding by smoking in FQ-AA association using three published approaches, and suggested that confounding by smoking is an unlikely explanation for the apparent association between FQ use and aortic aneurysm. These findings addressed a major limitation of epidemiologic studies regarding confounding by smoking due to an absence of data and strengthened the evidence for a FQ-related AA. Fourth, due to the limited number of included studies and limited data in the reports, we could not establish a safe window for FQ dose or duration, and it seems that higher doses or longer durations of FQ treatment exhibit stronger associations with aortic pathology (10, 13, 14). Finally, due to a lack of pharmacological interaction comparisons, we cannot determine whether FQs combined with other drugs, such as systemic corticosteroids and non-steroidal anti-inflammatory agents, increase the occurrence of aortic diseases (65).

## Conclusion

FQ use was associated with an increased short-term risk of developing AAD in the general population and a poor prognosis in patients who have been diagnosed with AAD. There are several important confounding factors from this study, which means that more data are required before this relationship is proven. Regardless, it can help physicians when considering prescribing FQs to patients with aortic dilatation and those at high risk of AAD. We propose considering an alternative antibiotic for patients with aortic pathology and avoiding this class of antibiotics in patients who are prone to AAD.

## Data Availability Statement

The original contributions presented in this study are included in the article/Supplementary material, further inquiries can be directed to the corresponding author.

## Author contributions

WF, DG, and BG proposed the conception and supervised the implementation process. CC and BG conducted the study search and screening, data extraction, and risk of bias assessment. CC, YL, and XL were responsible for data analysis. CC wrote the manuscript. BP, RS, ZC, and QL revised the manuscript. All authors reviewed and approved the final version of the work.
