# Supplementary material for: Do fluoroquinolones increase aortic aneurysm or dissection incidence and mortality? A systematic review and meta-analysis
Source: Front Cardiovasc Med. 2022 Aug 9;9:949538. doi: 10.3389/fcvm.2022.949538 (PMC9396038; doi:10.3389/fcvm.2022.949538)
Supplement: Supplementary file 1 [file Table_1.DOCX]

**Do fluoroquinolones increase aortic aneurysm or dissection incidence and mortality? a systematic review and meta-analysis**

Can Chen MD^1#^, Benjamin Patterson MD^2#^, Ruan Simpson MD^3^, Yanli Li MD^1^, Zhangzhang Chen MD^1^, Qianzhou Lv MD^1^, Daqiao Guo MD^4,5^, Xiaoyu Li PhD^1^, Weiguo Fu MD^4,5*^, Baolei Guo MD^4,5*^

**Affiliations:**

^1^Department of Pharmacy, Zhongshan Hospital, Fudan University, Shanghai, China

^2^Department of Vascular Surgery, University Hospital Southampton, Southampton, United Kingdom

^3^ Department of Pathology, Portsmouth Hospitals NHS Trust, United Kingdom

^4^Department of Vascular Surgery, Zhongshan Hospital, Institute of Vascular Surgery, Fudan University, Shanghai, China

^5^National Clinical Research Center for Interventional Medicine, Shanghai, China

# Can Chen and Benjamin Patterson contributed equally to this work and have shared first authorship.

**^*^Corresponding authors:**

Baolei Guo, Email: guo.baolei@zs-hospital.sh.cn, Department of Vascular Surgery, Zhongshan Hospital Fudan University, 180# Fengling Road, 200032, Shanghai, China, Tel: +86-21-64041990-2004;

Weiguo Fu, Email: fu.weiguo@zs-hospital.sh.cn, Department of Vascular Surgery, Zhongshan Hospital Fudan University, 180# Fengling Road, 200032, Shanghai, China, Tel: +86-21-64041990-2165.

Xiaoyu Li, Email: li.xiaoyu@zs-hospital.sh.cn, Department of Pharmacy, Zhongshan Hospital Fudan University, 180# Fengling Road, 200032, Shanghai, China, Tel: +86-21-64041990-5062.

**Running head** FQs and AAD

**Electronic Supplementary Material**

**Table of contents**

[Search strategy 3](#_Toc107941955)

[eTable 1 Reports excluded during full-text screening process 5](#_Toc107941956)

[Detailed study characteristics and risk of bias assessments (eTables S2-S12) 7](#_Toc107941957)

*[eTable 2 (](#_Toc107941958)*[Newton 2021](#_Toc107941958)^[17](#_Toc107941958)^[) 7](#_Toc107941958)

*[eTable 3(](#_Toc107941959)*[Gopalakrishnan 2020](#_Toc107941959)^[19](#_Toc107941959)^[) 8](#_Toc107941959)

*[eTable 4(](#_Toc107941960)*[Dong 2020](#_Toc107941960)^[18](#_Toc107941960)^[) 11](#_Toc107941960)

*[eTable 5(](#_Toc107941961)*[Lawaetz Kristensen 2021](#_Toc107941961)^[38](#_Toc107941961)^[) 13](#_Toc107941961)

*[eTable 6(](#_Toc107941962)*[Aspinall 2020](#_Toc107941962)^[37](#_Toc107941962)^[) 14](#_Toc107941962)

*[eTable 7(](#_Toc107941963)*[Maumus-Robert 2019](#_Toc107941963)^[16](#_Toc107941963)^[) 16](#_Toc107941963)

*[eTable 8(](#_Toc107941964)*[Pasternak 2018](#_Toc107941964)^[15](#_Toc107941964)^[) 18](#_Toc107941964)

*[eTable 9(](#_Toc107941965)*[Lee 2018](#_Toc107941965)^[14](#_Toc107941965)^[) 20](#_Toc107941965)

*[eTable 10(](#_Toc107941966)*[Lee 2015](#_Toc107941966)^[13](#_Toc107941966)^[) 22](#_Toc107941966)

*[eTable 11(](#_Toc107941967)*[Daneman 2015](#_Toc107941967)^[12](#_Toc107941967)^[) 24](#_Toc107941967)

*[eTable 12(](#_Toc107941968)*[Chen 2021](#_Toc107941968)^[23](#_Toc107941968)^[) 26](#_Toc107941968)

*[eTable 13(](#_Toc107941969)*[Chen 2022](#_Toc107941969)^[40](#_Toc107941969)^[) 28](#_Toc107941969)

*[eTable 14(](#_Toc107941970)*[Son 2022](#_Toc107941970)^[40](#_Toc107941970)^[) 29](#_Toc107941970)

[eTable 15 GRADE evidence profile for fluoroquinolones and risk of aortic aneurysm or aortic dissection 31](#_Toc107941971)

[eFigure 1 Forest plot of the risk of AA and AD in the comparison of FQs versus controls. 34](#_Toc107941972)

[eTable 16 Subgroup analyses of association of FQs with risk of AA/AD whin 30-day risk period 34](#_Toc107941973)

[eTable 17 Subgroup analyses of association of FQs with risk of AAD whin 60-day risk period 35](#_Toc107941974)

[eTable 18 Subgroup analyses of association of FQs with risk of AA/AD whin 90-day risk period 36](#_Toc107941975)

[eTable 19 Association between exposure to FQs and risk of AAD in individuals with infection 36](#_Toc107941976)

[eTable 20 Association between exposure to FQs and risk of mortality in patients with AAD 36](#_Toc107941977)

[eTable 21 Association between exposure to amoxicillin and risks of mortality in patients with AAD 37](#_Toc107941978)

[eTable 22 Association between exposure fluoroquinolones vs. exposure amoxicillin and the risks of mortality 37](#_Toc107941979)

[eTable 23 GRADE evidence profile for fluoroquinolones and mortality of AAD patients 37](#_Toc107941980)

[eTable 24 Association between FQs and mortality of AAD patients according to the type of aortic disease 38](#_Toc107941981)

### Search strategy

**The following databases were searched in** **March 2022**:

PubMed (1946 to March 31, 2022)

EMBASE (1974 to March 31, 2022)

The Cochrane Central Register of Controlled Trials (CENTRAL) (1947 to March 31, 2022)

Scopus (1960 to March 31, 2022)

Web of Science (1900 to March 31, 2022)

**Records identified through database searching:**

PubMed: 149

EMBASE: 991

The Cochrane Central Register of Controlled Trials (CENTRAL): 3

Scopus: 96

Web of Science: 195

**Electronic searches:**

**PubMed:**

(("Fluoroquinolones"[Mesh]) OR (((((((((((Fluoroquinolone*[Title/Abstract]) OR (Ciprofloxacin[Title/Abstract])) OR (Enoxacin[Title/Abstract])) OR (Enrofloxacin[Title/Abstract])) OR (Gatifloxacin[Title/Abstract])) OR (Gemifloxacin[Title/Abstract])) OR (Moxifloxacin[Title/Abstract])) OR (Norfloxacin[Title/Abstract])) OR (Ofloxacin[Title/Abstract])) OR (Levofloxacin[Title/Abstract])) OR (Pefloxacin[Title/Abstract]))) AND (((("Aneurysm"[Mesh]) OR (Aneurysm*[Title/Abstract])) OR (aortic dissection[Title/Abstract])) OR (Blood Vessel Dissection[Title/Abstract]))

**EMBASE:**

| No. | Query |
| --- | --- |
| #30 | #23 AND #28 AND [english]/lim |
| #29 | #23 AND #28 |
| #28 | #24 OR #25 OR #26 OR #27 |
| #27 | 'dissecting aneurysm':ab,kw,ti |
| #26 | 'aortic dissection':ab,kw,ti |
| #25 | 'aneurysm':ab,kw,ti |
| #24 | 'aneurysm'/exp |
| #23 | #1 OR #2 OR #3 OR #4 OR #5 OR #6 OR #7 OR #8 OR #9 OR #10 OR #11 OR #12 OR #13 OR #14 OR #15 OR #16 OR #17 OR #18 OR #19 OR #20 OR #21 OR #22 |
| #22 | 'fluoroquinolone*':ab,kw,ti |
| #21 | 'pefloxacin':ab,kw,ti |
| #20 | 'gemifloxacin':ab,kw,ti |
| #19 | 'gatifloxacin':ab,kw,ti |
| #18 | 'enrofloxacin':ab,kw,ti |
| #17 | 'enoxacin':ab,kw,ti |
| #16 | 'norfloxacin':ab,kw,ti |
| #15 | 'ofloxacin':ab,kw,ti |
| #14 | 'moxifloxacin':ab,kw,ti |
| #13 | 'ciprofloxacin':ab,kw,ti |
| #12 | 'levofloxacin':ab,kw,ti |
| #11 | 'pefloxacin'/exp |
| #10 | 'gemifloxacin'/exp |
| #9 | 'gatifloxacin'/exp |
| #8 | 'enrofloxacin'/exp |
| #7 | 'enoxacin'/exp |
| #6 | 'norfloxacin'/exp |
| #5 | 'ofloxacin'/exp |
| #4 | 'moxifloxacin'/exp |
| #3 | 'ciprofloxacin'/exp |
| #2 | 'levofloxacin'/exp |
| #1 | 'fluoroquinolone'/exp |

**The Cochrane Central Register of Controlled Trials (CENTRAL):**

ID Search

#1 MeSH descriptor: [Fluoroquinolones] explode all trees

#2 (Fluoroquinolone OR Fluoroquinolones OR Ciprofloxacin OR Enoxacin OR Enrofloxacin OR Gatifloxacin OR Gemifloxacin OR Moxifloxacin OR Norfloxacin OR Ofloxacin OR Levofloxacin OR Pefloxacin):ti,ab,kw

#3 #1 OR #2

#4 MeSH descriptor: [Aneurysm] explode all trees

#5 Aneurysm*:ti,ab,kw

#6 (Blood Vessel Dissection):ti,ab,kw

#7 (Aortic Dissection):ti,ab,kw

#8 #5 OR #6 OR #7

#9 #3 AND #8

**Scopus:**

((TITLE-ABS-KEY(Fluoroquinolones) OR TITLE-ABS-KEY(Ciprofloxacin) OR TITLE-ABS-KEY(Enoxacin) OR TITLE-ABS-KEY(Enrofloxacin) OR TITLE-ABS-KEY(Gatifloxacin) OR TITLE-ABS-KEY(Gemifloxacin) OR TITLE-ABS-KEY(Moxifloxacin) OR TITLE-ABS-KEY(Norfloxacin) OR TITLE-ABS-KEY(Ofloxacin) OR TITLE-ABS-KEY(Levofloxacin) OR TITLE-ABS-KEY(Pefloxacin))) AND ((TITLE-ABS-KEY(Aneurysm) OR TITLE-ABS-KEY("Aortic Dissection"))) AND ( LIMIT-TO ( LANGUAGE,"English" ) )

**Web of Science:**

#3 (#1) AND #2

#2 ((((((((((TS=(Fluoroquinolone*)) OR TS=(Ciprofloxacin)) OR TS=(Enoxacin)) OR TS=(Enrofloxacin)) OR TS=(Gatifloxacin)) OR TS=(Gemifloxacin)) OR TS=(Moxifloxacin)) OR TS=(Norfloxacin)) OR TS=(Ofloxacin)) OR TS=(Levofloxacin)) OR TS=(Pefloxacin)

#1 ((TS=(Aneurysm*)) OR TS=(Blood Vessel Dissection)) OR TS=(Aortic Dissection)

### eTable 1 Reports excluded during full-text screening process

| **No.** | **Reports** | **Reasons for excluding from the analysis** |
| --- | --- | --- |
| 1 | Yu, P. H., et al. (2020). The incidence of collagen-associated adverse events in pediatric population with the use of fluoroquinolones: A nationwide cohort study in Taiwan. BMC Pediatrics 20(1). | The patients included were pediatric population. |
| 2 | Sommet, A., et al. (2019). What Fluoroquinolones Have the Highest Risk of Aortic Aneurysm? A Case/Non-case Study in VigiBase®. J Gen Intern Med 34(4): 502-503. | The data for analysis were obtained from from VigiBase®. |
| 3 | Meng, L., et al. (2019). Assessing fluoroquinolone-associated aortic aneurysm and dissection: Data mining of the public version of the FDA adverse event reporting system. Int J Clin Pract 73(5): e13331 | The data for analysis were obtained from FDA adverse event reporting system. |
| 4 | Dolladille, C., et al. (2019). Fluoroquinolone and Aortic Dissection Is It a Class Effect? Journal of the American College of Cardiology 73(3): 382-383. | The data for analysis were obtained from from VigiBase®. |
| 5 | Zhang, M., et al. (2020). A quantitative bias analysis of the confounding effects due to smoking on the association between fluoroquinolones and risk of aortic aneurysm. Pharmacoepidemiology and Drug Safety 29(8): 958-961. | Not explore the association between FQs and AA/AD risk |
| 6 | Maumus-Robert, S., et al. (2020). Risk of Intracranial Aneurysm and Dissection and Fluoroquinolone Use A Case-Time-Control Study. Stroke 51(3): 994-997. | Not explore the association between FQs and AA/AD risk |
| 7 | Frankel, W. C., et al. (2019). Patients at risk for aortic rupture often exposed to fluoroquinolones during hospitalization. Antimicrobial Agents and Chemotherapy 63(2). | Not explore the association between FQs and AA/AD risk |
| 8 | Etminan, M., et al. (2019). Oral Fluoroquinolones and Risk of Mitral and Aortic Regurgitation. Journal of the American College of Cardiology 74(11): 1444-1450. | Not explore the association between FQs and AA/AD risk |
| 9 | Del Zotto, E. and A. Pezzini (2019). Use of fluoroquinolones and the risk of spontaneous cervical artery dissection. Eur J Neurol 26(7): 1028-1031. | Not explore the association between FQs and AA/AD risk |
| 10 | Lundstrom, K.-J., et al. (2021). Short-term ciprofloxacin prophylaxis for prostate biopsy and risk of aortic aneurysm. Nationwide, population-based cohort study. Scandinavian Journal of Urology 55(3): 221-226. | Use prostate biopsy as a proxy for exposure to FQs. |
| 11 | Newton, E. R., et al. (2021). Concerns about Study on Fluoroquinolone Use and Risk of Development of Aortic Aneurysm-Reply. JAMA Surgery. | Comment&Response |
| 12 | LeMaire, S. A. (2021). Fluoroquinolones in Patients With Aortic Aneurysms or Dissections: Pouring Gasoline on a Fire. J Am Coll Cardiol 77(15): 1888-1890. | Comment&Response |
| 13 | Lai, C. C., et al. (2021). Association of fluoroquinolones use with the risk of aortic aneurysm or aortic dissection: Facts and myths. Journal of Microbiology, Immunology and Infection 54(2): 182-184. | Comment&Response |
| 14 | Filiberto, A. C. and G. R. Upchurch (2021). Fluoroquinolones and Aortic Disease-Is It Time to Broaden the Warning? JAMA Surgery 156(3): 273. | Comment&Response |
| 15 | Aschenbrenner, D. S. (2021). Concerns About Fluoroquinolones and Aortic Aneurysm. The American journal of nursing 121(5): 24. | Comment&Response |
| 16 | Monti, M., et al. (2020). Letter to the Editor: Consideration on “An evaluation of reports of ciprofloxacin, levofloxacin, and moxifloxacin- association neuropsychiatric toxicities, long-term disability, and aortic aneurysms/dissections disseminated by the Food and Drug Administration and the European Medicines Agency” by Bennett et al. Expert Opinion on Drug Safety 19(8): 1055-1056. | Comment&Response |
| 17 | Gregory, S. H. (2019). Aortic Aneurysms and Fluoroquinolones: Is There a Link and Does it Matter in the Perioperative Period? Journal of Cardiothoracic and Vascular Anesthesia 33(8): 2123-2124. | Comment&Response |
| 18 | Upchurch, G. R., Jr. (2018). Evidence Against the Use of Fluoroquinolones in Patients With Aortic Pathology. JAMA Surg 153(9): e181988. | Comment&Response |
| 19 | Singh, S. and A. Nautiyal (2018). Do Fluoroquinolones Increase the Risk of Aortic Aneurysms and Aortic Dissection? J Am Coll Cardiol 72(12): 1379-1381. | Comment&Response |
| 20 | Terry, P. and D. P. Albon (2021). Every rose has its thorn: Fluoroquinolones and the prevalence of aortic dilation in non-cystic fibrosis bronchiectasis. American Journal of Respiratory and Critical Care Medicine 203(9). | Conference abstract |
| 21 | Son, N., et al. (2020). Risk of aortic aneurysm and aortic dissection with use of fluoroquinolones in Korea. Pharmacoepidemiology and Drug Safety 29(SUPPL 3): 471. | Conference abstract |
| 22 | Sylvain, N., et al. (2019). Fluoroquinolone adverse drug events: Results of an active surveillance project. Pharmacoepidemiology and Drug Safety 28: 350. | Conference abstract |
| 23 | Londhe, A. A., et al. (2019). A self controlled case series of fluoroquinolone exposure and the risk of aortic aneurysm or dissection. Pharmacoepidemiology and Drug Safety 28: 555. | Conference abstract |
| 24 | Campana, P., et al. (2020). Aortic rupture in patient on oral therapy with levofloxacin. Aging Clinical and Experimental Research 32(4): 755-757. | Case report |
| 25 | Zhang, M., et al. (2019). Confounding effect of smoking on the association between fluoroquinolone and risk of aortic aneurysm. Pharmacoepidemiology and Drug Safety 28: 306-307. | Duplicate |

### Detailed study characteristics and risk of bias assessments (eTables S2-S12)

### eTable 2 (Newton 2021^17^)

| **Study characteristics** | | |
| --- | --- | --- |
| Study type | Observational cohort study | |
| Countries | USA | |
| Interventions | Oral FQ | |
| Controls | Amox-clav, azithromycin, cephalexin, clindamycin, and SMX-TMP | |
| Definition of exposure to FQ | All prescription fills for an oral FQ in the database. | |
| Study period | January 1, 2005 to September 30, 2017 | |
| Data source | IBM MarketScan Commercial Database | |
| Population Enrolment criteria | All prescription fills for either an oral FQ or a comparator antibiotic between January 1, 2005, and September 30, 2017 (prior to US FDA warning), among adults aged 18 to 64 years were eligible.  Patients who had antibiotic episodes in which a prior antibiotic fill (FQ or comparator) or a preexisting diagnosis of aneurysm or dissection occurred within that 180-day lookback window were excluded; Individuals were also excluded if they were hospitalized within 30 days of their index fill date (including the day of their prescription fill) | |
| **Population characteristics** | | |
| Age | FQ group, median(IQR) 47 (36-57) years;  Control group, median(IQR) 43 (31-54) years | |
| Gender | FQ group, 58.7% males Control group, 40.5% males | |
| Number of patients | FQ group 7 338 704 patients, control group 24 284 910 patients | |
| **AAD characteristics** | | |
| Definition of AAD | Definition of AAD based on ICD-9-CM and ICD-10-CM codes. | |
| Number of AAD | AAD: FQ group, 6752; Control group, 17627  AA: FQ group, 5110; Control group, 13188  AD: FQ group, 368; Control group, 1088 | |
| Risk period | 90 days | |
| **Analysis strategy** | | |
| Analysis strategy | Inverse probability of treatment weighting (IPTW) in Cox proportional hazards regression was used to estimate the association of FQs compared with that of an antibiotic comparator. | |
| Confounding factors adjusted for | Age at fill, sex, Charlson Comorbidity Index components, other comorbidities, other medication use, region, indication for antibiotic use, and year and quarter of fill | |
| Additional notes | FQs were stratified by specific medication (ciprofloxacin, levofloxacin, and moxifloxacin) to assess whether the association was consistent across the entire fluoroquinolone class; interaction terms were used to assess potential effect measure modification across sex, age, and comorbidity; the authors removed subsequent antibiotic fills that occurred after an individual had an antibiotic episode when they received a diagnosis of aneurysm to prevent overestimation of risk after antibiotic exposure; and sensitivity analysis was performed after excluding patients with unknown indications. | |
| **Risk of bias assessment** | | |
| **Domain** | **Rating** | **Reasoning** |
| Study participation | Low | No concerns. All eligible patients in the database were enrolled. |
| Study attrition | Low | No concerns, no loss to follow-up or missing outcome data mentioned and data appears to be complete. |
| Prognostic factor measurement | Low | The authors were able to capture antibiotic fills but were unable to confirm that the prescriptions were consumed by the individual or taken as prescribed; however, they assume unused fills would be minimal and similar between cohorts. |
| Outcome measurement | Moderate | The definition of aneurysm relied on ICD-9-CM and ICD-10-CM codes. The authors were unable to capture undiagnosed aneurysms. In addition, abdominal imaging was not routinely performed. Thus, it is possible that aneurysms we classified as incident outcomes may have been preexisting, or that fluoroquinolones simply aggravated a preexisting condition rather than initiated a de novo aneurysm. |
| Study confounding | Moderate | Several risk factors associated with aneurysm development, such as smoking, were not captured (or reliably captured) in claims data. Smoking is an important risk factor in the development of aortic aneurysm. |
| Statistical analysis and reporting | Low | Adequate strategy and reporting of the adjusted analysis results. |
| Overall risk of bias | Moderate |  |

### eTable 3(Gopalakrishnan 2020^19^)

| **Study characteristics** | | |
| --- | --- | --- |
| **Study type** | Observational cohort study | |
| **Countries** | USA | |
| Intervention | Use of FQ | |
| Control | Use of azithromycin or SMX-TMP | |
| Definition of exposure to FQ | FQs exposure was defined as a prescription of FQs for a pneumonia or UTI patient; | |
| Study period | January 1, 2003 to September 30, 2015 | |
| Data source | IBM MarketScan Commercial Database | |
| Population Enrolment criteria | Patients aged 50 years or older with a diagnosis of pneumonia 3 days or less before initiating a FQ or azithromycin or patients aged 50 years or older with a UTI diagnosis 3 days or less before initiating a FQ or SMX-TMP were included.  Patients who had a diagnosis of AA/AD at baseline, alcohol or drug use disorder, hospitalizations in the 6 months before and including the day of cohort entry, and those who received antibiotics other than the study drugs (FQs or a comparator of interest) on the cohort entry date to enhance the comparability of the exposure groups and reduce confounding by indication were excluded. | |
| **Population characteristics** | | |
| **Age** | **UTIs cohort:**  FQ group, mean ±SD 62.07±10.36 years;  SMX-TMP group, mean ±SD 62.04±10.30 years  **Pneumonia cohort:**  FQ group, mean ±SD 63.68 ±10.93 years;  Azithromycin group, mean ±SD 63.63±10.92 years  **Amoxicillin cohort:**  FQ group, mean ±SD 60.55 ±9.34 years;  Amoxicillin group, mean ±SD 60.68±9.28 years | |
| **Gender** | **UTIs cohort:**  FQ group, 13.3% male SMX-TMP group, 13.0% male  **Pneumonia cohort:**  FQ group, 46.4% male  Azithromycin group, 46.3% male  **Amoxicillin cohort:**  FQ group, 44.0% male  Amoxicillin group, 44.0% male | |
| **Number of patients** | UTIs cohort: FQ 474 182 patients, comparator antibiotic 474 182 patients  Pneumonia cohort: FQ 139 772 patients, comparator antibiotic 139 772 patients | |
| **AAD characteristics** | | |
| Definition of AAD | Hospitalization with a primary discharge diagnosis of AAD based on ICD-9 code occurring in the 60 days following treatment initiation. | |
| Number of AAD | **UTIs cohort:**  AAD: FQ, 37; SMX-TMP, 35  AA: FQ, 33; SMX-TMP, 32  AD: FQ, 4; SMX-TMP, 3  **Pneumonia cohort:**  AAD: FQ, 35; Azithromycin, 13  AA: FQ, 29; Azithromycin, 10 AD: FQ, 6; Azithromycin, 3  **Amoxicillin**  AAD: FQ, 445; Amoxicillin, 284 | |
| Risk period | 60 days | |
| **Analysis strategy** | | |
| Analysis strategy | 1:1 propensity score matching was used to control for 85 potential confounders, which were measured during the 6 months before and including the cohort entry date (day of treatment initiation). Nearest-neighbor matching without replacement was performed using a caliper of 0.05 on the propensity score scale. Covariate balance within the matched cohorts was assessed using standardized differences,with a standardized difference less than 0.1 indicating adequate balance between groups. Hazard ratios and 95% CIs were estimated in each propensity score-matched cohort using Cox proportional hazards regression models. | |
| Confounding factors adjusted for | Demographics, calendar time, comorbidities, medication use, indicators of health care use as proxies for overall disease state, care intensity, and surveillance, as well as a validated, claims-based frailty index | |
| Additional notes | In sensitivity analyses, stratified Cox proportional hazards regression models using the matched sets was used as strata to account for the matching. | |
| **Risk of bias assessment** | | |
| **Domain** | **Rating** | **Reasoning** |
| Study participation | Low | No concerns. All eligible patients in the database were enrolled. |
| Study attrition | Low | No concerns. No loss to follow-up or missing outcome data mentioned and data appears to be complete. |
| Prognostic factor measurement | Low | No concerns. The authors were able to capture antibiotic prescription but were unable to confirm that FQs were consumed by the individual or taken as prescribed; however, they assume unused fills would be minimal and similar between cohorts. |
| Outcome measurement | Moderate | The authors used ICD-9 codes for the diagnosis of AA or AD, which is less accurate than clinical adjudication. Although these codes have not been validated in the data source. In addition, since they observed too few events to implement the imaging restriction in the pneumonia cohort, the observed findings could be affected by residual confounding or surveillance bias. |
| Study confounding | Low | The studies lacked accurate data on important risk factors, such as tobacco use or body mass index, and lacked data on other risk factors for AA/AD, such as family history or genetic predisposition. However, the authors do not anticipate these factors to be differential between users of fluoroquinolones and the comparator antibiotic. |
| Statistical analysis and reporting | Low | Adequate strategy and reporting of the adjusted analysis results. |
| Overall risk of bias | Low |  |

### eTable 4(Dong 2020^18^)

| **Study characteristics** | | |
| --- | --- | --- |
| Study type | Nested case-control study | |
| Countries | Taiwan, China | |
| Intervention | Use of FQs | |
| Control | Use of Amox-clav or Amp-sulb, extended-spectrum ceph | |
| Definition of exposure to FQ | At least a 3-day supply of antibiotics was available within the window (1 to 60 days before the index date) | |
| Study period | January 1, 2009 to November 30, 2015 | |
| Data source | Taiwan National Health Insurance Research Database | |
| Population Enrolment criteria | Patients aged 20 years or older were included;  We excluded patients with ambiguous sex information, history of AAD (ICD-9-CM), or any congenital disorders that potentially predisposed them to AAD. | |
| **Population characteristics** | | |
| Age | AAD 67.41±15.03 years  Matched controls 67.41±15.03 years | |
| Gender | AAD 71.37% males  Matched controls 71.37% males | |
| Number of patients | **Overall patients:**  AAD 28 948 patients, of which 5646 use FQ  Matched controls 289 480 patients, of which 29 635 use FQ  **Patients with indicated infections**  AAD 5391 patients, of which 200 use FQ  Matched controls 53880 patients, of which 1679 use FQ | |
| **AAD characteristics** | | |
| Definition of AAD | Diagnosis of AAD based on the ICD-9-CM codes. | |
| Number of aortic AAD | **60-day risk period**  200 AAD patients use FQ  221 AAD patients use Amox-clav or Amp-sulb  145 AAD patients use extended-spectrum ceph | |
| Risk period | 30 and 60 days | |
| **Analysis strategy** | | |
| Analysis strategy | For each case, 10 controls were randomly matched on birth year, sex, and follow-up duration from cohort entry to the index date.Two conditional logistic regression models to estimate the Odds Ratio and 95%CIs associated with FQs monotherapy compared with amox-clav or amp-sulb monotherapy and extended-spectrum ceph monotherapy; one model adjusted for the matching factors only, and the other adjusted for both matching factors and potential baseline confounders. | |
| Confounding factors adjusted for | Hypertension, ischemic heart disease, valve disorder, ischemic stroke, disorders of lipid metabolism, chronic obstructive pulmonary disease, chronic kidney disease, Charlson comorbidity score, tobacco smoking, claims-based frailty index, any episodes of infections, any use of fluoroquinolones, and any use of non-FQs antibiotics measured from cohort entry to 60 days before the index date. | |
| Additional notes | The study (1) evaluated a possible duration-response association for FQs; (2) mitigated potential spill over effects of FQs initiated before the risk window; (3) evaluated the effects of the choice of risk window and minimum antibiotic treatment duration; (4) reduced potential reverse causation between FQ use and AAD occurrence; (5) mitigated potential confounding due to infection severity by classifying antibiotic use by treatment setting, dosage form, and cephalosporin generation; (6) examined potential effect measure modification by infection type and patient characteristic; (7) examined whether the risk varied by AAD subtype; (8) examined the effect of the operational definition of AAD; and (9) mitigated the concern about misdiagnosis of AA as LRTIs or GUTIs. | |
| **Risk of bias assessment** | | |
| **Domain** | **Rating** | **Reasoning** |
| Study participation | Low | No concerns. All eligible patients in the database were enrolled. |
| Study attrition | Low | No concerns. No loss to follow-up or missing outcome data mentioned and data appears to be complete. |
| Prognostic factor measurement | Low | No concerns. Exposure to fluoroquinolone was identified by a reimbursement code, but could not be determined the medication adherence for one patient due to lacking of relevant data. However, we considered that this impact was small and did not significantly change the results. |
| Outcome measurement | Moderate | Although ICD-9 code used in the study a validated outcome algorithm, it could not rule out the possibility of outcome misclassification because coding practice may vary by country. |
| Study confounding | Low | The residual confounding between antibiotic use and AA/AD due to tobacco smoking, frailty, or other unmeasured confounders could bias the observed association owing to improper adjustment for an intermediate variable. However, the bias to would lead to a greater effect of infections and not an attenuation of effect as observed in the analysis. |
| Statistical analysis and reporting | Low | Adequate strategy and reporting of the adjusted analysis results. |
| Overall risk of bias | Low |  |

### eTable 5(Lawaetz Kristensen 2021^38^)

| **Study characteristics** | | |
| --- | --- | --- |
| Study type | Case-crossover study | |
| Countries | Denmark | |
| Interventions | Use of FQ | |
| Controls | No use of FQ | |
| Definition of exposure to FQ | Exposure to FQ within a window was defined by the occurrence of a redeemed FQ prescription within that window. | |
| Study period | 1996 to 2016 | |
| Data source | The Danish National Patient Register, the Danish National Prescription Register, the Danish Civil Registration System and the Danish National Register of Causes of Death | |
| Population Enrolment criteria | The study included everyone diagnosed with a ruptured aortic aneurysm with known exposure to FQ during the 24 weeks before the index date.  The study excluded persons with any history of Marfan syndrome or Ehlers-Danlos syndrome and persons surviving more than 30 days after the diagnosis of the ruptured aortic aneurysm without undergoing any surgery within the first week. | |
| **Patients characteristics** | | |
| Age | FQ group, median(IQR) 77 (70-81) years;  No FQ group, median(IQR) 77 (71-82) years | |
| Gender | FQ group, 69.0% males No FQ group, 66.5% males | |
| Number of patients | NA | |
| **AAD characteristics** | | |
| Definition of AAD | Cases were all patients diagnosed with a ruptured thoracic aneurysm, ruptured thoracoabdominal aneurysm or ruptured abdominal aortic aneurysm | |
| Number of aortic AAD | FQs (within 28 d) 58 patients, No FQs (within 28 d) 188 patients | |
| Risk period | 28, 60 and 90 days | |
| **Analysis strategy** | | |
| Analysis strategy | Conditional logistic regression was used to estimate the odds ratio (OR) associating the use of FQ with ruptured AA. Both crude and the adjusted ORs are presented with a 95% confidence interval (CI). | |
| Confounding factors adjusted for | NR | |
| Additional notes | The main result was analysed in subgroups defined by sex, age, year of diagnosis and type of ruptured AA.  The hazard period of 28 days in the main analysis was stratified by the redemption of the prescription 0-14 days and 15-28 days before rupture. The hazard period was set to 60 and 90 days instead of 28 days as used in the main analysis. Besides, Another series of four control periods were added one year before the index date to eliminate seasonal variation risk. | |
| **Risk of bias assessment** | | |
| **Domain** | **Rating** | **Reasoning** |
| Study participation | Moderate | Period of patient’s recruitment and method used to identify population were not adequately described. |
| Study attrition | Low | No concerns. No loss to follow-up or missing outcome data mentioned and data appears to be complete. |
| Prognostic factor measurement | Low | Exposure to FQ was defined by the occurrence of a redeemed FQ prescription. However, this could not be determined the medication adherence for one patient due to lacking of relevant data. However, we considered that this impact was small and did not significantly change the results. |
| Outcome measurement | Moderate | The authors identified the AA/AD patients based on ICD-10 codes. although ICD-10 codes is a validated outcome algorithm, it could not rule out the possibility of outcome misclassification because coding practice may vary by country. There is no imaging data to assist in the identification of aortic aneurysms and increase the detection rate of aneurysms. |
| Study confounding | Moderate | An increased risk of rupture by FQ use could be confounded by indications since the actual infection could cause the outcome. Confounding by indication could also be at large in the study. |
| Statistical analysis and reporting | Low | Adequate strategy and reporting of the adjusted analysis results. |
| Overall risk of bias | Moderate |  |

### eTable 6(Aspinall 2020^37^)

| **Study characteristics** | | | |
| --- | --- | --- | --- |
| Study type | Self-controlled case series analysis study | | |
| Countries | USA | | |
| Interventions | Use of oral FQs (levofloxacin, ciprofloxacin, moxifloxacin) | | |
| Controls | Use of amoxicillin or amox-clav (ie amoxicillin group), azithromycin, doxycycline, cefuroxime/cephalexin, SMX-TMP, or no antibiotics | | |
| Definition of exposure to FQ | Receiving at least one outpatient prescription for a FQ within antibiotic risk periods. | | |
| Study period | October 1, 2014 to September 30, 2018 | | |
| Data source | The National Patient Care Database, The Pharmacy Benefits Management (PBM) Services outpatient prescription database (v 3.0), Corporate Data Warehouse Health Factors data, the Vital Status file | | |
| Population Enrolment criteria | Patients aged ≥18 years who had the outcomes of AAD based on ICD-9/10-CM codes in the primary or principal position for emergency department visits or hospitalizations, respectively, or death during the study period and received oral FQs as outpatients in this same time frame were included.  Patients who received >42 consecutive days of FQs (ie chronic therapy) were excluded. | | |
| **Population characteristics** | | | |
| Age | FQ group, mean±SD 68.6 ±8.8 years;  Control group, not report. | | |
| Gender | FQ group, 98.3% male Control group, not report. | | |
| Number of patients | NA | | |
| **AAD characteristics** | | | |
| Definition of AAD | Definition of AAD based on ICD-9/10-CM codes. | | |
| Number of AAD | **30-day risk period**  FQs 124 patients, amoxicillin 32 patients, azithromycin 17 patients, cefuroxime/  cephalexin 18 patients, doxycycline 13 patients, SMX-TMP 27 patients, other antibiotics 29 patients, no antibiotics 1874 patients  **60-day risk period**  FQs 192 patients, amoxicillin 60 patients, azithromycin 27 patients, cefuroxime/  cephalexin 27 patients, doxycycline 20 patients, SMX-TMP 37 patients, other antibiotics 35 patients, no antibiotics 1673 patients | | |
| Risk period | 30 and 60 days | | |
| **Analysis strategy** | | | |
| Analysis strategy | Conditional Poisson regression models were used to estimate the association between FQs and each of the outcomes vs the antibiotics of interest using within person comparisons. Results are presented as adjusted incidence rate ratios (aIRRs) and 95% confidence intervals. | | |
| Confounding factors adjusted for | Time-varying covariates including age, fiscal year of index date, and common respiratory (ie pneumonia, chronic obstructive pulmonary disease exacerbation, bronchitis, pharyngitis, sinusitis, cough, upper respiratory infection), urinary (ie urinary tract infection, pyelonephritis, prostatitis, bacteriuria), and skin and soft-tissue (ie cellulitis, skin abscess, diabetic foot infection, skin and soft tissue) infections associated with receipt of outpatient antibiotics. | | |
| Additional notes | Sensitivity analyses was conducted by running the Poisson regression models after removing patients who had >1 outcome of the same type and examining a 10-day risk period for all outcomes and 60 days for AAD. | | |
| **Risk of bias assessment** | | | |
| **Domain** | **Rating** | | **Reasoning** |
| Study participation | Moderate | | The authors did not clarify whether the study excluded patients with history of AAD, or any congenital disorders that potentially predisposed them to AAD. |
| Study attrition | Low | | No concerns. No loss to follow-up or missing outcome data mentioned and data appears to be complete. |
| Prognostic factor measurement | Low | | Exposure to FQ was defined as the occurrence of a FQ prescription. However, this could not be determined the medication adherence for one patient due to lacking of relevant data. However, we considered that this impact was small and did not significantly change the results. |
| Outcome measurement | Moderate | | The authors identified the AAD patients based on ICD-10 or ICD-9 codes. although ICD-10 codes or ICD-9 is a validated outcome algorithm, it could not rule out the possibility of outcome misclassification because coding practice may vary by country. There is no imaging data to assist in the identification of aortic aneurysms and increase the detection rate of aneurysms. |
| Study confounding | Moderate | Although fixed confounders are controlled for in an SCCSA, and the author included important time-varying covariates, residual time-varying confounding is still possible such as severity of infection. | |
| Statistical analysis and reporting | Low | Adequate strategy and reporting of the adjusted analysis results. | |
| Overall risk of bias | Moderate |  | |

### eTable 7(Maumus-Robert 2019^16^)

| **Study characteristics** | | |
| --- | --- | --- |
| Study type | Case-time-control analyses study | |
| Countries | France | |
| Interventions | Use of FQ | |
| Controls | Use of amoxicillin | |
| Definition of exposure to FQ | FQ exposures were identified from out-hospital reimbursements. | |
| Study period | July 1, 2010 to December 31, 2015 | |
| Data source | French health insurance nationwide databases (SNIIR-AM) | |
| Population Enrolment criteria | Patients who aged 18 years or older with incident aortoiliac aneurysm or dissection, and had been exposed to FQs (or amoxicillin) during the 180 days before the date of outcome occurrence (i.e., index date); and were not hospitalized in the 180 days before the outcome (no information on in-hospital antibiotic exposure available from the database). | |
| **Population characteristics** | | |
| Age | FQ group, median(IQR) 70 (62-80) years;  Amoxicillin group, median(IQR) 67 (57-79) years | |
| Gender | FQ group, 36.0% female Amoxicillin group, 25.3% female | |
| Number of patients | FQs 946 patients, Amoxicillin 3476 patients | |
| **AAD characteristics** | | |
| Definition of AAD | Definition of AAD based on ICD-10 codes and medical procedures. | |
| Number of AAD | FQs group: exposed cases 36 patients in 30-risk window; exposed cases 48 patients in 60-risk window; exposed cases 65 patients in 90-risk window, 23 patients in control window;  Amoxicillin group: exposed cases 82 patients in 30-risk window; exposed cases 132 patients in 60-risk window; exposed cases 180 patients in 90-risk window, 161 patients in control window; | |
| Risk period | 30, 60 and 90 days | |
| **Analysis strategy** | | |
| Analysis strategy | Trend for exposure along the period was controlled using a reference group of randomly selected subjects free of the event who were individually matched to cases regarding age and sex (up to 10 per case).  The conditional logistic model were used to estimate the association. Results are presented as adjusted odds ratios (ORs) and 95% confidence intervals. Association with amoxicillin use, considered a marker of a potential indication bias, was studied similarly. | |
| Confounding factors adjusted for | Time-varying confounders including the use of other antibiotics, corticosteroids, anticoagulant agents, antiplatelet agents, and antihypertensive drugs. | |
| Additional notes | Sensitivity analyses were conducted: 1) extending the risk window (and corresponding control windows) to 60 or 90 days; and 2) extending the population to patients with history of hospitalization during the 180 days prior to index date. A complementary case-control analysis considering cases and ≤10 randomly selected control subjects matched on a disease risk score was conducted, which also explored the association with fluoroquinolone past use (day 61 to day 365 before the event). | |
| **Risk of bias assessment** | | |
| **Domain** | **Rating** | **Reasoning** |
| Study participation | Moderate | The basic characteristics of the included population are not fully presented. |
| Study attrition | Low | No concerns. No loss to follow-up or missing outcome data mentioned and data appears to be complete. |
| Prognostic factor measurement | Low | Exposure to fluoroquinolone was identified by a reimbursement code, but could not be determined the medication adherence for one patient due to lacking of relevant data. However, we considered that this impact was small and did not significantly change the results. |
| Outcome measurement | Moderate | The authors identified the AA/AD patients based on ICD-10 codes. although ICD-10 codes is a validated outcome algorithm, it could not rule out the possibility of outcome misclassification because coding practice may vary by country. There is no imaging data to assist in the identification of aortic aneurysms and increase the detection rate of aneurysms. |
| Study confounding | Low | No concerns. Clear definitions of the important confounders were provided and measured appropriately. |
| Statistical analysis and reporting | Low | Adequate strategy and reporting of the adjusted analysis results. |
| Overall risk of bias | Moderate |  |

### eTable 8(Pasternak 2018^15^)

| **Study characteristics** | | |
| --- | --- | --- |
| Study type | Historical cohort study | |
| Countries | Sweden | |
| Interventions | Use of FQ | |
| Controls | Use of amoxicillin | |
| Definition of exposure to FQ | Receiving a prescription for FQs or amoxicillin during the study period. | |
| Study period | July 2006 to December 2013 | |
| Data source | National Prescribed Drug Register, National Patient Register, Statistics Sweden, Swedish Cause of Death Register | |
| Population Enrolment criteria | Patients who received a prescription for FQs or amoxicillin during the study and who were aged 50 years or older. Besides, the included patients should have no previous diagnosis of AAD, were not admitted to hospital in the previous 120 days (information on in hospital antibiotic exposure was not available), did not receive multiple antibiotics (any) on the same day, did not have a diagnosis indicating end stage illness or drug/alcohol misuse, and had used at least one prescription drug in the past year. | |
| **Patients characteristics** | | |
| Age | FQ group, mean± SD 67.9±10.8 years;  Amoxicillin group, mean± SD 68.0±10.4 years | |
| Gender | FQ group, 45.0% males Amoxicillin group, 45.0% males | |
| Number of patients | FQs 360088 episodes, Amoxicillin 360088 episodes | |
| **AAD characteristics** | | |
| Definition of AAD | A first diagnosis of AAD based on ICD-10 codes. | |
| Number of AAD | FQs group: 64 cases in the 60-day risk period;  Amoxicillin group: 40 cases in the 60-day risk period; | |
| Risk period | 60 days | |
| **Analysis strategy** | | |
| Analysis strategy | Cox proportional hazards regression, with days since start of treatment as the time scale, was used to estimate the hazard ratio for AAD, comparing episodes of fq and amoxicillin use. The proportional hazards assumption were assessed by measuring the interaction between treatment status and time scale using a Wald test. | |
| Confounding factors adjusted for | To control for potential confounders, treatment episodes of FQs and amoxicillin use were matched in a 1:1 ratio on the basis of propensity scores using the 5->1 digit greedy matching algorithm. The propensity score for FQs exposure was estimated by a logistic regression model, including 47 covariates as predictors, covering demographic information, medical history, prescription drug use, and healthcare use. The standardised difference less than 10% were considered a covariate to be well balanced. | |
| Additional notes | Subgroup analyses were conducted according to sex and age.  Sensitivity analyses were conducted by restricting the outcome to cases of AAD with rupture or dissection alone (not without rupture or dissection); associated with admission to hospital alone (not emergency department) or in which AAD were the underlying cause of death; and involving patients who had had aortic surgery or died within 30 days of the date of diagnosis or in whom AAD were the underlying cause of death. | |
| **Risk of bias assessment** | | |
| **Domain** | **Rating** | **Reasoning** |
| Study participation | Low | No concerns. All eligible patients in the database were enrolled. |
| Study attrition | Low | No concerns. No loss to follow-up or missing outcome data mentioned and data appears to be complete. |
| Prognostic factor measurement | Moderate | The study was relied on filled prescriptions to define drug exposure; nonadherence to fluoroquinolones would bias results towards the null. |
| Outcome measurement | Moderate | The authors identified the AA/AD patients based on ICD-10 codes. although ICD-10 codes is a validated outcome algorithm, it could not rule out the possibility of outcome misclassification because coding practice may vary by country. There is no imaging data to assist in the identification of aortic aneurysms and increase the detection rate of aneurysms. |
| Study confounding | Moderate | Several risk factors associated with aneurysm development, such as smoking, were not captured (or reliably captured) in claims data. Smoking is an important risk factor in the development of aortic aneurysm. |
| Statistical analysis and reporting | Low | Adequate strategy and reporting of the adjusted analysis results. |
| Overall risk of bias | Moderate |  |

### eTable 9(Lee 2018^14^)

| **Study characteristics** | | |
| --- | --- | --- |
| Study type | Case-crossover and case-time-control study | |
| Countries | Taiwan, China | |
| Interventions | Use of FQ | |
| Controls | No use of FQ | |
| Definition of exposure to FQ | Exposure to FQs was identified by a reimbursement code of oral FQs with a prescription length of 3 days or more. | |
| Study period | 2002 to 2011 | |
| Data source | Longitudinal Health Insurance Database (LHID) (based on the year 2000 Version) | |
| Population Enrolment criteria | All inpatients diagnosed with AA or AD based on ICD-9-CM codes plus diagnostic evidence obtained by advanced imaging studies such as angiography, transesophageal or transthoracic echocardiography, thoracic or abdominal computed tomography, or magnetic resonance imaging were included. | |
| **Popualtion characteristics** | | |
| Age | Overall AA/AD patients, mean± SD 70.58 ±13.77 years;  Disease risk score-matched controls patients without AA/AD, mean± SD 70.48±13.69 years. | |
| Gender | Overall AA/AD patients, 72.46% males  Disease risk score-matched controls patients without AA/AD, 72.46% males. | |
| Number of patients | NR | |
| **AAD characteristics** | | |
| Definition of AAD | Patients with aortic aneurysm or dissection were identified based on ICD-9-CM codes plus diagnostic evidence obtained by  advanced imaging studies such as angiography, transesophageal or transthoracic echocardiography, thoracic or abdominal computed tomography, or magnetic resonance imaging. | |
| Number of aortic AAD | Case-crossover study design:  Of 1213 cases in the hazard period, 19 use FQs;  Of 1213 cases in the referent period, 7 use FQs.  Control-crossover study design:  Of 1213 cases in the hazard period, 9 use FQs;  Of 1213 cases in the referent period, 12 use FQs. | |
| Risk period | 60, 120 and 180 days | |
| **Analysis strategy** | | |
| Analysis strategy | In the main analysis, the authors compared exposure to FQs between the hazard period and 1 randomly selected referent period, using either the case-crossover or control-crossover analysis. Odds ratios (ORs) for AA/AD associated with the use of FQs and 95%CIs were calculated by conditional logistic regression. The case-time-control ORs were determined by dividing the case-crossover OR by the control-crossover OR. | |
| Confounding factors adjusted for | Adjusted for age, gender, index year, comorbidities, healthcare utilization, use of medications.  A disease-risk score-matched time control analysis was performed to investigate the potential time-trend bias. | |
| Additional notes | In the sensitivity analysis, the authors repeated the main analysis using a 1:5 ratio of hazard period to referent period, to adjust for the effect of time-variant confounders; shortened or extended the length of hazard periods from 60 days to either 120 or 180 days; performed a duration-response analysis(<3 days, 3 to 14 days, and >14 days); and estimated the number needed to harm (NNH) for AAD. | |
| **Risk of bias assessment** | | |
| **Domain** | **Rating** | **Reasoning** |
| Study participation | Low | No concerns. All eligible patients in the database were enrolled. |
| Study attrition | Low | No concerns. No loss to follow-up or missing outcome data mentioned and data appears to be complete. |
| Prognostic factor measurement | Low | There is no information on adherence to prescriptions in the database. Although we cannot exclude the possibility of misclassification of exposure. It is likely that such exposure misclassification was random, not biasing the results. |
| Outcome measurement | Low | No concerns. Patients with aortic aneurysm or dissection were identified based on ICD-9-CM codes plus diagnostic evidence obtained by  advanced imaging studies such as angiography, transesophageal or transthoracic echocardiography, thoracic or abdominal computed tomography, or magnetic resonance imaging. |
| Study confounding | Low | No concerns. Clear definitions of the important confounders were provided and measured appropriately. |
| Statistical analysis and reporting | Low | Adequate strategy and reporting of the adjusted analysis results. |
| Overall risk of bias | Low | **Rating:** Low risk of bias |

### eTable 10(Lee 2015^13^)

| **Study characteristics** | | |
| --- | --- | --- |
| Study type | Nested case-control study | |
| Countries | Taiwan, China | |
| Interventions | Use of FQ | |
| Controls | No use of FQ | |
| Definition of exposure to FQ | Use of FQ was assumed whenever there was any order for a reimbursement code of oral fluoroquinolone with a prescription length of 3 days or longer. | |
| Study period | January 2000 to December 2011 | |
| Data source | National Health Insurance Research Database (NHIRD) of Taiwan. | |
| Population Enrolment criteria | Patients who aged 18 years or older were included. | |
| **Population characteristics** | | |
| Age | AA/AD mean ±SD 74.17±11.7/66.2±14.5 years;  Controls mean ±SD 71.0±13.7 years. | |
| Gender | AA/AD 74.1%/71.5% males;  Controls 72.9% males. | |
| Number of patients | AA/AD 1477 patients;  Controls 147700 patients | |
| **AAD characteristics** | | |
| Definition of AAD | Patients with AAD were identified based on ICD-9-CM codes plus the use of advanced imaging such as thoracic or abdominal computed tomography, transesophageal or transthoracic echocardiography, magnetic resonance imaging, or angiography. | |
| Number of AAD | AA/AD 1477 patients, and not report the number of patients treated with FQs  Controls 147700 patients, and not report the number of patients treated with FQs | |
| Risk period | 60 and 61-365 days | |
| **Analysis strategy** | | |
| Analysis strategy | Under a time-matched case-control sampling scheme, the odds ratios estimate the rate ratios (RRs). Incidence RRs of AAD (plus 95%CIs) were estimated by using conditional logistic regression analysis adjusted for all covariates.  In addition to conventional multivariate analysis,we constructed a propensity score for adjustment and matching. Using the entire cohort, the authors derived the propensity score using a logistic regression model that included all potential predictors for fluoroquinolone therapy. Propensity score matching was conducted using a greedy matching algorithm without any trimming. | |
| Confounding factors adjusted for | age, gender, calendar year, Charlson index score, cardiovascular disease related, risk factors, drugs use. | |
| Additional notes | Duration response analyses were performed by testing the linear association between the ordinal categories of drug use duration and the risk of incident AAD. We also performed subgroup analyses in high-risk patients.  Subgroups analysis were conducted by sex and age. | |
| **Risk of bias assessment** | | |
| **Domain** | **Rating** | **Reasoning** |
| Study participation | Moderate | The authors did not clarify whether the study excluded patients with history of AA/AD, or any congenital disorders that potentially predisposed them to AA/AD. |
| Study attrition | Low | No concerns. No loss to follow-up or missing outcome data mentioned and data appears to be complete. |
| Prognostic factor measurement | Low | There is no information on adherence to prescriptions in the database. Although we cannot exclude the possibility of misclassification of exposure. It is likely that such exposure misclassification was random, not biasing the results. |
| Outcome measurement | Low | No concerns. Patients with aortic aneurysm or dissection were identified based on ICD-9-CM codes plus diagnostic evidence obtained by advanced imaging studies such as angiography, transesophageal or transthoracic echocardiography, thoracic or abdominal computed tomography, or magnetic resonance imaging. |
| Study confounding | Moderate | The study lacked data on lifestyle factors, including smoking, alcohol drinking, and body mass index, which could bias the results. |
| Statistical analysis and reporting | Low | Adequate strategy and reporting of the adjusted analysis results. |
| Overall risk of bias | Moderate |  |

Current use refers to patients having a fluoroquinolone prescription filled within 60 days of the index date;

Past use refers to patients having a prescription filled between 61 and 365 days prior to the index date;

Any use in prior year refers to having a fluoroquinolone prescription that was filled for 3 or more days in the 1-year period before the index date.

### eTable 11(Daneman 2015^12^)

| **Study characteristics** | | |
| --- | --- | --- |
| Study type | Longitudinal cohort study | |
| Countries | Canada | |
| Interventions | Use of FQ | |
| Controls | No use of FQ | |
| Definition of exposure to FQ | FQ prescriptions were measured in the database which records medications prescribed to older patients. | |
| Study period | April 1 1997 to March 31 2012 | |
| Data source | Ontario Registered Persons Database | |
| Population Enrolment criteria | Patients who turning age 65 years during the study period were included.  Patients who remained less than 65 years throughout the study interval or the rare patients with a missing health card identifier under universal healthcare were excluded. | |
| **Population characteristics** | | |
| Age | Use of FQ 65 years;  No use of FQ 65 years | |
| Gender | Use of FQ 48.6% males;  No use of FQ 48.9% males. | |
| Number of patients | Use of FQ 657950 patients;  No use of FQ 1086410 patients | |
| **AAD characteristics** | | |
| Definition of AAD | Patients with aortic aneurysm were identified based on ICD-9 and ICD-10 codes. | |
| Number of AAD | NR | |
| Risk period | 30 days | |
| **Analysis strategy** | | |
| Analysis strategy | Multivariate Cox proportional hazards model to allow coefficients to differ for the primary predictor (FQ exposure) as well as for other risk factors. Owing to the large cohort size and lengthy longitudinal follow-up, multivariable models were performed on a 50% random subset of the study cohort and confirmed by replication on the remaining 50% subset of the cohort. | |
| Confounding factors adjusted for | Demographic risk factors included sex and income quintile; prior healthcare utilisation included total hospital admissions and physician visits in the year prior to enrolment; comorbidities of interest included prior urinary tract infections, prior pneumonia, diabetes mellitus, hypertension, atherosclerosis (coronary artery disease, cerebrovascular disease, or peripheral vascular disease), chronic kidney disease, chronic obstructive pulmonary disease, malignancy, liver disease, inflammatory bowel disease, hypothyroidism and depression. | |
| Additional notes | Sensitivity analysis was performed by limiting to only those patients who received at least one FQ prescription during follow-up; those aortic aneurysm events coded as primary diagnoses; and emergency admissions for aortic aneurysm events.  Subgroup analyses were performed by stratifing by baseline patient characteristics, and also assessed the association separately for ciprofloxacin (the most common FQ prescribed) compared to other FQs.  To test the specificity of the findings, the authors tested for an absence of an association between amoxicillin prescriptions and the same adverse events (negative tracer exposure). To ensure the fidelity of the methods, the authors further tested the anticipated presence of an association between FQs and the risk of Clostridium difficile infection (positive tracer outcome).  The number of preventable aortic aneurysms in the cohort was estimated if the numbers or durations of FQ prescriptions were reduced by 50%. | |
| **Risk of bias assessment** | | |
| **Domain** | **Rating** | **Reasoning** |
| Study participation | Moderate | The authors did not clarify whether the study excluded patients with history of AA/AD, or any congenital disorders that potentially predisposed them to AA/AD. |
| Study attrition | Low | No concerns. No loss to follow-up or missing outcome data mentioned and data appears to be complete. |
| Prognostic factor measurement | Low | Misclassification of fluoroquinolone exposure is possible, and we cannot be sure that patients complied with treatment. Bias caused by patient exposure to fluoroquinolone might affect results |
| Outcome measurement | Moderate | The outcome events of the study were multicausal (eg, tendon ruptures from osteonecrosis or trauma, retinal detachments from globe injuries, and aortic aneurysms from hypertension) which might dilute our estimates of relative hazard. |
| Study confounding | Moderate | The authors cannot rule out residual confounding, since the neutral tracer (amoxicillin) was not entirely null. |
| Statistical analysis and reporting | Low | Adequate strategy and reporting of the adjusted analysis results. |
| Overall risk of bias | Moderate |  |

### eTable 12(Chen 2021^23^)

| **Study characteristics** | | |
| --- | --- | --- |
| Study type | Cohort study | |
| Countries | Taiwan of China | |
| Interventions | Use of FQ | |
| Controls | No use of FQ or use amoxicillin | |
| Definition of exposure to FQ | FQs prescriptions were measured in the database which records medications prescribed to older patients. | |
| Study period | January 1, 2001 to December 31, 2013 | |
| Data source | Taiwan’s National Health Insurance Research Database | |
| Population Enrolment criteria | Patients who were admitted for aortic disease (AD or AA) were identified among the entire Taiwanese population.  Patients who were younger than 20 years, had previously been diagnosed with any aortic disease, and died during admission were excluded. | |
| **Population characteristics** | | |
| Age | Use of FQ Median(IQR) 73.1 (63.4 to 79.9) years; No use of FQ Median(IQR) 68.5 (55.7 to 77.3) years;  Use of amoxicillin Median(IQR) 67.2 (55.2 to 76.0) years; No use of amoxicillin Median(IQR) 68.7 (55.9 to 77.4) years | |
| Gender | Use of FQ 69.7% males; No use of FQ 72.0% males.  Use of amoxicillin 74.5% males; No use of amoxicillin 71.9% males. | |
| Definition of AAD | The ICD-9-CM diagnostic codes were used to identify patients with aortic disease (ICD-9-CM: 441.0 for AD and 441.1 to 441.9 for AA) at admission. | |
| Number of patients (with pre-existing AAD) | Use of FQ 14,853 patients; No use of FQ 682,318 patients;  Use of amoxicillin 28,026 patients; No use of amoxicillin 669,145 patients. | |
| **Outcomes characteristics** | | |
| Number of outcomes | All cause death FQs exposure period 817, control period 13,863; amoxicillin exposure period 449, control period 14,231;  Aortic death FQs exposure period 128, control period 2174; amoxicillin exposure period 75, control period 2,227;  Aortic open surgery FQs exposure period 119, control period 3350; amoxicillin exposure period 178, control period 3,291;  Aortic stent FQs exposure period 78, control period 1740; amoxicillin exposure period 91, control period 1,727. | |
| Definition of outcomes | All cause death was defined by a withdrawal from the National Health Insurance program.  Aortic death was determined by examining the cause of death in the main diagnosis in the discharge records for inpatient hospital deaths, the primary diagnosis of the last emergency department visit, or the cause of hospitalization within 7 days of death for out-of-hospital deaths.  The occurrence of aortic open surgery and aortic stent was detected by using National Health Insurance reimbursement codes. | |
| Risk period | 60 days | |
| **Analysis strategy** | | |
| Analysis strategy | The associations between FQs or amoxicillin use and the risk of outcome were investigated by using univariable and multivariable time-dependent Cox proportional hazards models. | |
| Confounding factors adjusted for | Adjusted for age, sex, socioeconomic status (monthly income and urbanization level), medical utilization in the previous year (number of outpatient visits, emergency department visits, and hospitalization), 14 medical conditions or comorbidities, Charlson comorbidity index score, hospital level for the index admission, whether the patient underwent aortic surgery at the index admission, 9 types of antihypertensive agents within 3 months after discharge, the number of antihypertensive agents, 5 types of other medications within 3 months after discharge, and prescription for FQs or amoxicillin 2 months before the index admission. | |
| Additional notes | Subgroup analyses were stratified by the type of aortic disease (AA or AD) and whether the patient underwent surgery during the index admission.  Negative control exposure (amoxicillin) and 3 negative outcomes(fracture, trauma, and stroke) to detect the possibility of residual confounding. | |
| **Risk of bias assessment** | | |
| **Domain** | **Rating** | **Reasoning** |
| Study participation | Low | No concerns. The sampling frame and recruitment are adequately described, and the methods to identify the AA/AD population is sufficiently valid to limit potential bias. |
| Study attrition | Low | No concerns. No loss to follow-up or missing outcome data mentioned and data appears to be complete. |
| Prognostic factor measurement | Low | Misclassification of fluoroquinolone exposure is possible; however, such exposure misclassification was random, and any effect on the results would be limited. |
| Outcome measurement | Low | A clear definition of outcome is provided, including duration of follow-up. The method of outcome identification used is adequately valid and reliable to limit misclassification bias. |
| Study confounding | Low | Although some residual confounding may have been caused by unknown confounders such as computed tomography results, blood pressure, body mass index, and smoking history. However, the authors used amoxicillin as a negative control exposure to limit confounding factors associated with prescription of antibiotics, and obtained null findings. This demonstrated the robust nature of the results. |
| Statistical analysis and reporting | Low | Adequate strategy and reporting of the adjusted analysis results. |
| Overall risk of bias | Low |  |

### eTable 13(Chen 2022^40^)

| **Study characteristics** | | |
| --- | --- | --- |
| Study type | Cohort study | |
| Countries | Taiwan of China | |
| Interventions | Use of FQ | |
| Controls | first-, or second-generation cephalosporins. | |
| Definition of exposure to FQ | FQs prescriptions were measured in the database which records medications prescribed to older patients. | |
| Study period | 2000 to 2017 | |
| Data source | Taiwan’s National Health Insurance Research Database | |
| Population Enrolment criteria | Patients who received a primary diagnosis of urinary tract infections.  Patients who were younger than 18 years, who received both fluoroquinolones and  cephalosporins, and who had AA or AD within 180 days before the index date were excluded. | |
| **Population characteristics** | | |
| Age | FQ group: 18-29 years (9.20%); 30-44 years (14.45%); 45-59 years (21.41%); 60-74 years(25.65%); ≥75 years (29.29%)  First-or second-generation cephalosporins group: 18-29 years (9.90%); 30-44 years (14.78%); 45-59 years (21.08%); 60-74 years(25.45%); ≥75 years (28.79%) | |
| Gender | FQ group 28.2% males;  First-or second-generation cephalosporins group 28.1% males | |
| Number of patients | FQ group 28 568 patients;  First-or second-generation cephalosporins group 28 568 patients | |
| **AAD characteristics** | | |
| Definition of AAD | Patients with aortic aneurysm were identified based on ICD-9 codes. | |
| Number of patients | FQ group 20;  First-or second-generation cephalosporins group 22 | |
| Risk period | 90 days | |
| **Analysis strategy** | | |
| Analysis strategy | The associations between FQs or first-or second-generation cephalosporins use and the risk of AAD were investigated by using univariable and multivariable time-dependent Cox proportional hazards models. | |
| Confounding factors adjusted for | adjusted by the covariates including sex, age, urbanization, unit type, marital status, education level, baseline hospitalized stays, baseline comorbidities, and baseline medication | |
| Additional notes | A subgroup analysis and test of interaction effects were performed to evaluate the effect of sex and age on different stratifications through multivariable Cox regression. Moreover, a landmark analysis was conducted to assess the potential time-varying  effect of antibiotics on AA and AD during different periods, namely 0 to 12, 0 to 3, 3 to 6, and 6 to 12 months after the index date | |
| **Risk of bias assessment** | | |
| **Domain** | **Rating** | **Reasoning** |
| Study participation | Low | No concerns. The sampling frame and recruitment are adequately described, and the methods to identify the AA/AD population is sufficiently valid to limit potential bias. |
| Study attrition | Low | No concerns. No loss to follow-up or missing outcome data mentioned and data appears to be complete. |
| Prognostic factor measurement | Moderate | Misclassification of fluoroquinolone exposure is possible. |
| Outcome measurement | Low | A clear definition of outcome is provided, including duration of follow-up. The method of outcome identification used is adequately valid and reliable to limit misclassification bias. |
| Study confounding | Moderate | Although some residual confounding may have been caused by unknown confounders such as alcohol consumption, body mass index, and smoking history. |
| Statistical analysis and reporting | Low | Adequate strategy and reporting of the adjusted analysis results. |
| Overall risk of bias | Moderate |  |

### eTable 14(Son 2022^40^)

| **Study characteristics** | | |
| --- | --- | --- |
| Study type | Nested case-control study | |
| Countries | Korea | |
| Interventions | Use of FQ | |
| Controls | No use of FQ | |
| Definition of exposure to FQ | The exposure of interest was the use of a fluoroquinolone in the year prior to the index date. | |
| Study period | 2013 to 2017 | |
| Data source | National Health Insurance Service | |
| Population Enrolment criteria | Patients aged 40 to 99 years 2014-2017 in the NHIS database.  We excluded patients who had taken FQs more than once during the year prior  to the cohort entry date, were diagnosed with AA/AD or underlying related diseases during the year prior to the cohort entry date. | |
| **Population characteristics** | | |
| Age | AAD: 40-49 years (7%); 50-59 years (14.9%); 60-69 years (23.7%); 70-79 years(32%); 80-89 years(19.4%); ≥90 years (3%)  Matched controls: 40-49 years (7%); 50-59 years (14.9%); 60-69 years (23.7%); 70-79 years(32%); 80-89 years(19.4%); ≥90 years (3%) | |
| Gender | AAD 62.5%  Matched controls 62.5% | |
| Number of patients | AAD 29638 patients, of which 8562 use FQ  Matched controls 118552 patients, of which 25387 use FQ | |
| **AAD characteristics** | | |
| Definition of AAD | Patients with aortic aneurysm were identified based on ICD-10 codes. | |
| Number of patients | AAD 29638 patients  Matched controls 118552 patients | |
| Risk period | 60 days | |
| **Analysis strategy** | | |
| Analysis strategy | The associations between FQs use and the risk of AAD were investigated by using multivariate conditional logistic regression analysis.. | |
| Confounding factors adjusted for | Adjusted for sex, age, underlying disease, Charlson comorbidity index, medication use, history of procedure/surgery | |
| Additional notes | Subgroup analyses were condcuted by sex and age.  Sensitivity analyses were condcuted by changing the definition of AA/AD occurrence. | |
| **Risk of bias assessment** | | |
| **Domain** | **Rating** | **Reasoning** |
| Study participation | Low | No concerns. The sampling frame and recruitment are adequately described, and the methods to identify the AA/AD population is sufficiently valid to limit potential bias. |
| Study attrition | Low | No concerns. No loss to follow-up or missing outcome data mentioned and data appears to be complete. |
| Prognostic factor measurement | Moderate | Misclassification of fluoroquinolone exposure is possible. |
| Outcome measurement | Low | A clear definition of outcome is provided, including duration of follow-up. The method of outcome identification used is adequately valid and reliable to limit misclassification bias. |
| Study confounding | Moderate | Although some residual confounding may have been caused by unknown confounders such as blood pressure, smoking status,  and family history. |
| Statistical analysis and reporting | Low | Adequate strategy and reporting of the adjusted analysis results. |
| Overall risk of bias | Moderate |  |

### eTable 15 GRADE evidence profile for fluoroquinolones and risk of aortic aneurysm or aortic dissection

| **Outcome** | **Number of studies** | **Quality assessment** | | | | | **Effect ^c^** | | **Certainty of evidence** |
| --- | --- | --- | --- | --- | --- | --- | --- | --- | --- |
|  |  | **Risk of bias** | **Inconsistency** | **Indirectness** | **Imprecision** | **Other concerns ^b^** | **No. of AA/AD events with FQs** | **Relative effect estimate (95% CI)** |  |
| **1 Risk of AA/AD within 30-day risk period** | | | | | | | | | |
| 1.1 FQs vs. no FQ ^l^ | 5 | Serious ^d^ | Serious ^d^ | Serious ^f^ | Not serious ^g^ | None | > 418 **^a^** | 1.42 (1.11 to 1.81) | Very low |
| 1.1.1 FQs vs. amoxicillin | 2 | Serious ^d^ | Not serious | Not serious | Not serious ^g^ | None | 160 | 1.52 (1.17 to 1.96) | Moderate |
| 1.1.2 FQs vs. amox-clav or amp-sull | 1 | Not serious | Not serious | Not serious | Serious ^g^ | None | 200 | 1.11 (0.89 to 1.38) | Moderate |
| 1.1.3 FQs vs. azithromycin | 1 | Serious ^d^ | Not serious | Not serious | Not serious | None | 124 | 2.15 (1.27 to 3.64) | Moderate |
| 1.1.4 FQs vs. cephalosporins | 2 | Not serious ^d^ | Not serious | Not serious | Serious ^g^ | None | 324 | 1.35 (0.81 to 2.25) | Moderate |
| 1.1.5 FQs vs. doxycycline | 1 | Serious ^d^ | Not serious | Not serious | Serious ^g^ | None | 124 | 1.81 (1.00 to 3.28) | Low |
| 1.1.6 FQs vs. SMX-TMP | 1 | Serious ^d^ | Not serious | Not serious | Serious ^g^ | None | 124 | 0.81 (0.53 to 1.24) | Low |
| 1.1.7 FQs vs. no antibiotics | 1 | Serious ^d^ | Not serious | Not serious | Not serious | None | 124 | 1.64 (1.33 to 2.02) | Moderate |
| **2 Risk of AA/AD within 60-day risk period** | | | | | | | | | |
| 2.1 FQs vs. no FQ ^l^ | 7 | Serious ^d^ | Serious ^d^ | Serious ^f^ | Not serious ^g^ | None | 1098 | 1.44 (1.22 to 1.71) | Low |
| 2.1.1 FQs vs. amoxicillin | 4 | Not serious | Not serious | Not serious | Not serious ^g^ | None | 749 | 1.50 (1.31 to 1.71) | High |
| 2.1.2 FQs vs. amox-clav or amp-sulb | 1 | Not serious | Not serious | Not serious | Serious ^g^ | None | 200 | 1.01 (0.82 to 1.24) | Moderate |
| 2.1.3 FQs vs. azithromycin | 2 | Not serious | Not serious | Not serious | Not serious | None | 227 | 2.13  (1.50 to 3.02) | High |
| 2.1.4 FQs vs. cephalosporins | 2 | Not serious | Not serious | Not serious | Serious ^g^ | None | 392 | 1.06 (0.69 to 1.62) | Moderate |
| 2.1.5 FQs vs. doxycycline | 1 | Serious ^d^ | Not serious | Not serious | Not serious ^g^ | None | 192 | 1.68 (1.04 to 2.71) | Moderate |
| 2.1.6 FQs vs. SMX-TMP | 2 | Not serious | Not serious | Not serious | Serious ^g^ | None | 229 | 0.91 (0.68 to 1.22) | Moderate |
| 2.1.7 FQs vs. no antibiotics | 2 | Serious ^d^ | Not serious | Not serious | Not serious | None | 192 | 1.58 (1.42 to 1.77) | Moderate |
| **3 Risk of AA/AD within 90-day risk period** | | | | | | | | | |
| 3.1 FQs vs. no FQ ^l^ | 3 | Serious ^d^ | Not serious | Serious ^f^ | Not serious ^g^ | None | 6875 | 1.47 (1.24 to 1.75) | Low |
| 3.1.1 FQs vs. amoxicillin | 1 | Serious ^d^ | Not serious | Not serious | Not serious ^g^ | None | 65 | 1.59  (1.19 to 2.12) | Moderate |
| 3.1.1 FQs vs. cephalosporins | 1 | Serious ^d^ | Not serious | Not serious | Not serious ^g^ | None | 20 | 0.88 (0.47 to 1.65) | Moderate |
| 3.1.1 FQs vs. other antibiotics ^i^ | 1 | Serious ^d^ | Not serious | Serious ^h^ | Not serious ^g^ | None | 6752 | 1.20 (1.17 to 1.23) | Low |

Summary of findings including results and GRADE evidence profile for fluoroquinolones and risk of AA/AD. Of note, observational studies start with an overall high rating in GRADE for risk of AA/AD.

Abbreviations: FQs: Fluoroquinolones; CI: confidence interval; NR: not report; GRADE: Grading of Recommendation, Assessment, Development and Evaluation approach; SMX-TMP: combined trimethoprim and sulfamethoxazole.

^a^ A study of Daneman et al included in the meta-analysis did not report the number of AA/AD events with FQs, thus, we failed to count the exact total number of events.

^b^ We planned to assess publication bias by visual inspection of funnel plots where more than 10 studies were included; this was not done as less than 10 studies were included for each outcome, however, we have no reason to suspect publication bias here.

^c^ Estimates contain combined estimates of both hazard, risk, and odds ratios.

^d^ Rated down for risk of bias as a moderate risk of bias study were included in this meta-analysis.

^e^ Some inconsistency was present, which seemed to be explained by the differences in how the AA/AD was defined in the included studies.

^f^ No FQ referred that other antibiotics or no antibiotics were used in the included studies, and the relative risk of AA/AD compared to a specific antibiotic cannot be obtained by this comparison.

^g^ Confidence intervals crossed the null and/or the lower boundary and upper boundary probably lead to different decisions in clinical practice.

^h^ Other antibiotics used in the included studies are not necessarily representative of all of the antibiotics.

^i^ Other antibiotics referred to amoxicillin-clavulanate, azithromycin, cephalexin, clindamycin, and sulfamethoxazole trimethoprim.

^j^ The five studies were divided into 11 comparisons.

^k^ The seven studies were divided into 15 comparisons.

^l^ No FQ include other antibiotics or no antibiotics.


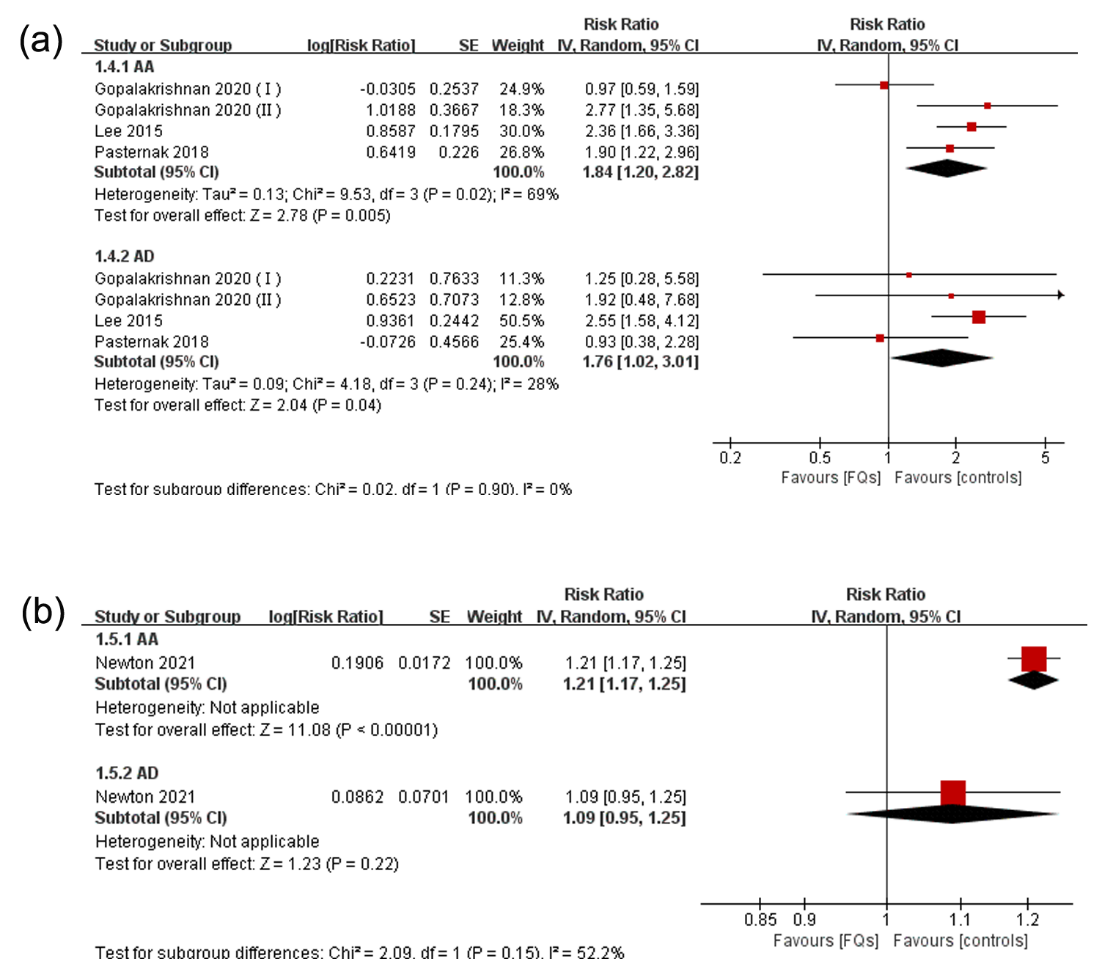


eFigure 1 Forest plot of the risk of AA and AD in the comparison of FQs versus controls. **(a) within a 60-day risk period; (b) within a 90-day risk period. FQs, FQs, fluoroquinolones; AA, aortic aneurysm; AD, aortic dissection; IV: inverse variance; CI, confidence interval.**

### eTable 16 Subgroup analyses of association of FQs with risk of AA/AD whin 30-day risk period

| **Subgroup** | **Studies (n)** | **Risk Ratio (95% CI)** | **P value between groups** |
| --- | --- | --- | --- |
| **Study type** |  |  | <0.001 |
| Cohort studies^12^ | 1 | 2.24(2.02, 2.48) |  |
| Nest case-control studies^18^ | 1 | 1.02(0.85, 1.22) |  |
| Self-control studies^16,37,38^ | 3 | 1.46(1.23, 1.73) |  |
| **Age(years)** |  |  | NA |
| ≥ 65^12,38^ | 2 | 1.78 (1.09, 2.92) |  |
| **Sex** |  |  | 0.19 |
| Male^12,38^ | 2 | 1.87(1.22, 2.86) |  |
| Female^38^ | 1 | 1.17(0.67, 2.04) |  |
| **With ruptured or without ruptured AA** |  |  | NA |
| Ruptured AA or AD^12,16,38^ | 3 | 2.10(1.22, 3.60) |  |
| **Anatomical site** |  |  | 0.13 |
| AAA^38^ | 1 | 1.30(0.91, 1.86) |  |
| TAAA^38^ | 1 | 3.64(1.30, 10.19) |  |
| TAA^38^ | 1 | 0.86(0.24, 3.08) |  |

Abbreviation: FQs, fluoroquinolones; AA, aortic aneurysm; AD, aortic dissection; AAA, abdominal aortic aneurysm; TAA, thoracic aortic aneurysm; TAAA, thoracoabdominal aortic aneurysm; CI, confidence interval, NA, not applicable; NR, not report.

### eTable 17 Subgroup analyses of association of FQs with risk of AAD whin 60-day risk period

| **Subgroup** | **Studies (n)** | **Risk Ratio (95% CI)** | **P value between groups** |
| --- | --- | --- | --- |
| **Study type** |  |  | 0.64 |
| Cohort studies^15,19^ | 2 | 1.56(0.96, 2.56) |  |
| Nest case-control studies^13,18,39^ | 3 | 1.34(0.95, 1.89) |  |
| Self-control studies^14,16,37,38^ | 4 | 1.64(1.30, 2.06) |  |
| **Age (**years) |  |  | 0.43 |
| < 65^15^ | 1 | 1.58(0.61, 4.09) |  |
| ≥ 65^15,18^ | 2 | 1.07(0.77, 1.48) |  |
| **Sex** |  |  | 0.18 |
| Male^13,15,18^ | 3 | 1.25(0.90, 1.75) |  |
| Female^13,15^ | 2 | 1.69(1.27, 2.26) |  |
| **With ruptured or without ruptured AA** |  |  | 0.58 |
| Ruptured AA or AD^15,16,18,19^ | 4 | 1.40(0.99, 2.00) |  |
| Unruptured AA^18,19^ | 2 | 1.22(0.87, 1.72) |  |
| **Anatomical site** |  |  | 0.23 |
| AAA^18,19^ | 2 | 1.33(0.85, 2.06) |  |
| TAAA^19^ | 1 | 1.23(0.53, 2.86) |  |
| TAA^18^ | 1 | 0.82(0.57, 1.18) |  |

Abbreviation: FQs, fluoroquinolones; AA, aortic aneurysm; AD, aortic dissection; AAD, aortic aneurysm or aortic dissection; AAA, abdominal aortic aneurysm; TAA, thoracic aortic aneurysm; TAAA, thoracoabdominal aortic aneurysm; CI, confidence interval, NA, not applicable.

### eTable 18 Subgroup analyses of association of FQs with risk of AA/AD whin 90-day risk period

| **Subgroup** | **Studies (n)** | **Risk Ratio (95% CI)** | **P value between groups** |
| --- | --- | --- | --- |
| **Study type** |  |  | 0.005 |
| Cohort studies^17,40^ | 2 | 1.20 (1.17, 1.23) |  |
| Self-control studies^16,38^ | 2 | 1.90 (1.38, 2.62) |  |
| **Age(years)** |  |  | NA |
| < 65^17^ | 1 | 1.20 (1.17, 1.23) |  |
| **Sex** |  |  | 0.79 |
| Male^17^ | 1 | 1.22(1.18, 1.26) |  |
| Female^17^ | 1 | 1.21(1.15, 1.27) |  |
| **Anatomical site** |  |  | <0.001 |
| AAA^17^ | 1 | 1.31(1.25, 1.37) |  |
| TAAA^17^ | 1 | 0.90(0.68, 1.19) |  |
| TAA^17^ | 1 | 1.05(0.98, 1.13) |  |

Abbreviation: FQs, fluoroquinolones; AA, aortic aneurysm; AD, aortic dissection; AAA, abdominal aortic aneurysm; TAA, thoracic aortic aneurysm; TAAA, thoracoabdominal aortic aneurysm; CI, confidence interval, NA, not applicable; NR, not report.

### eTable 19 Association between exposure to FQs and risk of AAD in individuals with infection

| **Outcome** | **Studies (n)** | **Risk Ratio (95% CI)** | **P value** |
| --- | --- | --- | --- |
| 30-day risk period | 2 | 1.30 (1.04, 1.63) | 0.02 |
| 60-day risk period | 3 | 1.31 (1.08, 1.59) | 0.005 |
| 90-day risk period | 2 | 1.21 (1.10, 1.33) | 0.0001 |

### eTable 20 Association between exposure to FQs and risk of mortality in patients with AAD

| **Outcome** | **FQs exposure period** | | **Control exposure period** | | **Risk Ratio (95% CI)** | **P value** |
| --- | --- | --- | --- | --- | --- | --- |
|  | **No. of events** | **Person-year** | **No. of events** | **Person-year** |  |  |
| All-cause mortality | 817 | 213327.3 | 13863 | 107031.9 | 1.61 (1.50-1.73) | <.001 |
| Aortic-specific mortality | 128 | 2344.5 | 2174 | 107483.6 | 1.80 (1.50-2.15) | <.001 |

### eTable 21 Association between exposure to amoxicillin and risks of mortality in patients with AAD

| **Outcome** | **Amoxicillin exposure period** | | **Control exposure period** | | **Risk Ratio (95% CI)** | **P value** |
| --- | --- | --- | --- | --- | --- | --- |
|  | **No. of events** | **Person-year** | **No. of events** | **Person-year** |  |  |
| All-cause mortality | 449 | 4485.5 | 14231 | 104873.7 | 0.95 (0.86-1.04) | 0.31 |
| Aortic-specific mortality | 75 | 4491.5 | 2227 | 105336.6 | 0.99 (0.79-1.15) | 0.93 |

### eTable 22 Association between exposure fluoroquinolones vs. exposure amoxicillin and the risks of mortality

| **Outcome** | **FQ exposure period** | | **Amoxicillin exposure period** | | **Risk Ratio (95% CI)** | **P value** |
| --- | --- | --- | --- | --- | --- | --- |
|  | **No of events** | **Person-year** | **No of events** | **Person-year** |  |  |
| All-cause mortality | 782 | 2167.6 | 414 | 4325.8 | 1.73 (1.52-1.96) | <.001 |
| Aortic-specific mortality | 121 | 2184.4 | 68 | 4331.4 | 1.99 (1.44-2.75) | <.001 |

### eTable 23 GRADE evidence profile for fluoroquinolones and mortality of AAD patients

| **Outcome** | **Number of studies** | **Quality assessment** | | | | | **Effect ^c^** | | **Certainty of evidence** |
| --- | --- | --- | --- | --- | --- | --- | --- | --- | --- |
|  |  | **Risk of bias** | **Inconsistency** | **Indirectness** | **Imprecision** | **Other concerns ^b^** | **No. of events with FQs** | **Relative effect estimate (95% CI)** |  |
| **1 All-cause** **mortality** | | | | | | | | | |
| 1.1 FQs vs. no FQ ^d^ | 1 | Not serious | Not serious | Serious **^a^** | Not serious | None | 817 | 1.61 (1.50 to 1.73) | Moderate |
| 1.2 FQs vs. amoxicillin | 1 | Not serious | Not serious | Serious **^a^** | Not serious | None | 782 | 1.73 (1.52 to 1.96) | Moderate |
| **2 Aortic-specific mortality** | | | | | | | | | |
| 2.1 FQs vs. no FQ ^d^ | 1 | Not serious | Not serious | Serious **^a^** | Not serious | None | 128 | 1.80 (1.50 to 2.15) | Moderate |
| 2.2 FQs vs. amoxicillin | 1 | Not serious | Not serious | Serious ^a^ | Not serious | None | 121 | 1.99 (1.44 to 2.75) | Moderate |

Summary of findings including results and GRADE evidence profile for fluoroquinolones and risk of mortality in AAD. Of note, observational studies start with an a

Abbreviations: FQs: Fluoroquinolones; CI: confidence interval; GRADE: Grading of Recommendation, Assessment, Development and Evaluation approach.

^a^ The study included was a population-based study conducted in East Asia (Taiwan), and the characteristics and incidence of aortic disease and antibiotics prescription may differ from those of other populations, thus, the results may not be directly generalizable to other patient population.

^b^ We planned to assess publication bias by visual inspection of funnel plots where more than 10 studies were included; this was not done as less than 10 studies were included for each outcome, however, we have no reason to suspect publication bias here.

^c^ Estimates contain combined estimates of both hazard, risk, and odds ratios.

^d^ No FQ include other antibiotics or no antibiotics.

### eTable 24 Association between FQs and mortality of AAD patients according to the type of aortic disease

| Outcome | Risk Ratio (95% CI) | | *P* value between groups |
| --- | --- | --- | --- |
|  | AD patients | AA patients |  |
| All-cause mortality | 1.57 (1.37-1.80) | 1.63 (1.50-1.77) | 0.985 |
| Aortic-specific mortality | 1.85 (1.33-2.57) | 1.79 (1.44-2.22) | 0.746 |

Abbreviation: FQs, fluoroquinolones; AA, aortic aneurysm; AD, aortic dissection;
